# Effects of Physiological Status and Environmental Factors on the Lure Responses of Three Pest Fruit Fly Species (Diptera: Tephritidae)

**DOI:** 10.1007/s10886-024-01516-8

**Published:** 2024-07-08

**Authors:** Tania Pogue, Kevin Malod, Christopher W. Weldon

**Affiliations:** 1 Department of Zoology and Entomology, Forestry and Agricultural Biotechnology Institute, University of Pretoria, Private Bag X20, Hatfield, 0028 South Africa; 2 Department of Conservation Ecology and Entomology, Faculty of AgriSciences, Stellenbosch University, Stellenbosch, South Africa

**Keywords:** Attractants, Lure response, Monitoring, Motivational state, *Bactrocera*, *Ceratitis*

## Abstract

Biotic and abiotic factors influence how insects respond to stimuli. This can make it challenging to interpret captures in traps used to monitor pest abundance in management programmes. To address this, the lure response of three pest fruit flies (Diptera: Tephritidae) was evaluated in a semi-field setting with respect to several physiological and environmental factors. Using standardised methods with known fly numbers in field cages, the response to Biolure (food-based lure) was evaluated for *Ceratitis capitata, Ceratitis cosyra* and *Bactrocera dorsalis.* Response to the male lures was tested: E.G.O PheroLure for *C. capitata* and *C. cosyra*, Trimedlure for *C. capitata*, and methyl eugenol for *B. dorsalis*. The physiological variables evaluated were fly age, sex, weight, and total body nutritional composition. The environmental effects of temperature, relative humidity and light intensity were also assessed. Protein-deprived adults responded more strongly to Biolure. The response to Biolure was not sex-specific. Fly age influenced the response of all species to all tested lures. However, this effect was species and lure specific. Temperature was the most influential environmental factor, with response generally increasing with temperature. Lower thresholds for lure response, despite the proximity of responsive flies, range from 12.21 to 22.95 °C depending on the species and lure tested. These results indicate that trapping systems and management activity thresholds must take physiological and environmental variation into account to increase their accuracy.

**Supplementary Information:**

The online version contains supplementary material available at 10.1007/s10886-024-01516-8.

## Introduction

Trapping is a crucial component of fruit fly (Diptera: Tephritidae) management programmes. Traps can be used to monitor populations, detect and delimit invasions, and verify pest status (Enkerlin and Reyes-Flores 2018). Beyond population monitoring, traps are also used to control fruit fly populations through attract-and-kill approaches. Trap catches are converted to “flies per trap per day” (FTD) to standardize variability in the number of traps and the capture period used (Enkerlin and Reyes-Flores 2018). FTD is used as a population index and action threshold to guide fruit fly control decisions, and is calculated as the total number of flies trapped in an area divided by the number of traps that were inspected and the number of days the traps were active. However, trapping levels do not solely rely on pest abundance but also on environmental conditions and the responsiveness of the target species to the attractant used (Díaz-Fleischer et al. 2014).

Physiological state has a direct impact on motivation level and resource orientated behaviours (Browne 1993), which is directly linked to fruit fly trap response. The volatile chemicals used as fruit fly attractants are associated with food, oviposition or mating resources to manipulate resource-findings behaviours (Díaz-Fleischer et al. 2014). Diaz-Fleischer et al. (2009) found that *Anastrepha ludens* and *(A) obliqua* fed a nutritionally deficient diet responded more to food-based lures using protein odours than when fed sugar or fruit. They also found that food-based (protein odours) lure response differed by species, age, and sex (Díaz-Fleischer et al. 2009). In *Bactrocera cucurbitae* (Coquillett) females, physiological state affected the response to both food-based (protein) and ovipositional (host volatile) odours (Vargas et al. 2018). Although physiological state influences the response of some *Anastrepha* species to protein-based food lures, it does not influence the response of all *Anastrepha* species to host volatiles that are used as oviposition sites (Diaz-Santiz et al. 2016; López-Ley et al. 2016). In the Queensland fruit fly, *Bactrocera tryoni* (Froggatt), responsiveness to the parapheromone cuelure also relates feeding status through its effects on the rate of sexual maturation, which improved response by males at younger ages (Weldon et al. 2008). Olfactory response by *(B) tryoni* can also decline significantly by age, with decreases in attraction starting at 15 and 6 weeks of age for males and females respectively (Tasnin et al. 2020). Mating status in the females of several *Anastrepha* species impacts their response to olfactory cues, with unmated females responding stronger to male sex pheromones than their mated counterparts (Córdova-García et al. 2021). Mating status elicits a similar response by female Mediterranean fruit flies, *Ceratitis capitata* (Wiedemann) (Jang et al. 1999). In the context of behavioural responses to traps, this implies that male pheromone-based baits are more effective for catching unmated females (Lima et al. 2001). Robacker (1998) even found that irradiation, that is usually used for the sterile insect technique, had an effect on lure response in fruit flies. Clearly, the effect of physiology on resource-related behaviours is complex and species-specific. This in turn makes correlating lure responses to fly physiology challenging. Due to this, a better understanding of these interactions is needed to improve attractant design in future and improve the accuracy of population estimates gathered from the use of fruit fly lures.

The role that environmental conditions play in tephritid lure response is understudied and relatively poorly understood compared with the role that physiological state plays (Aluja et al. 2011; Díaz-Fleischer et al. 2014). Field studies that include the impact of abiotic variables on tephritid trap response are often contradictory. Cunningham et al. (1978) reported that the oriental fruit fly, *Bactrocera dorsalis* (Hendel), and *C. capitata* respond more to baited traps in drier conditions (reduced rainfall and low relative humidity). However, Vayssières et al. (2009) show that trap capture probability for *B. dorsalis* increases with relative humidity. In contrast, there was no relationship between rainfall (and associated relative humidity) and trap capture in the Mexican fruit fly, *Anastrepha ludens* Loew (Aluja et al. 1996). Some studies have found that trap captures for *B. dorsalis* are positively correlated with temperature (Bana et al. 2017; Patel et al. 2013), whereas another found trap captures decreased for *B. dorsalis* at higher temperatures (Adzim et al. 2016).

Environmental variables, such as temperature and relative humidity, can also have an effect on the attractants used in traps. Release rates of trimedlure increase with temperature, although this is dependent on the dispenser type used (Domínguez-Ruiz et al. 2008; Flores et al. 2017). Suckling et al. (2008), found that the release rate of methyl eugenol plug dispensers was negatively correlated with humidity only in the cooler testing site. The effects of weather on lure release rates and efficiency are often confounded by interactions between abiotic variables (such as that between temperature and humidity) (Gómez-Escobar et al. 2022). Few studies have been conducted on the effects of environmental variables on lure release rates and trap performance (Shelly and Cloonan 2023). Clearly, there is a need for more research on the effects that environmental variables have on tephritid lure response.

This study investigated the effects of weather and physiological state on the performance of several attractants for tephritid species under semi-field conditions, with a focus on species that are of economic concern. This included *C. capitata*, the marula fruit fly, *Ceratitis cosyra* (Walker), and *B. dorsalis* (Hendel). Several commercially used fruit fly attractants were tested, namely BioLure, E.G.O PheroLure, trimedlure and methyl eugenol. We also related the change in weight of lure formulations when placed in traps with temperature and relative humidity under field conditions. It was hypothesized that flies, regardless of species, would show a female-bias in their response to the food-based lure BioLure. Additionally, it was predicted that the availability of protein in the diet, and subsequent higher whole body protein content, would reduce the response to BioLure, and that flies with access to protein and approaching sexual maturity would show a higher response to male lures (E.G.O PheroLure, trimedlure, methyl eugenol). Overall, higher temperatures should result in increased fly activity and lure response. It was further predicted that overall lure response would decrease at higher relative humidity, due to suspected limitations on lure volatilisation. By quantifying the relationship between physiological state, the environment and fruit fly lure response, it is hoped that the precision and accuracy of fruit fly population estimates can be improved. In turn this could improve fruit fly management decisions in the field.

## Methods and Materials

### Fly Husbandry

For all species, experimental flies were obtained from pre-established cultures at the University of Pretoria. Fly cultures were maintained in a climate-controlled room at approximately 23.0 ± 3.6 °C and 40–60% relative humidity. A 14:10 light-dark photoperiod was maintained in the climate room with a one-hour dawn and dusk period simulated for the first and last hour of the light cycle. The main day lighting comprised of a combination of 20 W (G5, Eurolux, Sandton, South Africa) and 58 W (58 W’840, Osram, Germany) fluorescent tubes. The dawn and dusk lighting comprised of 8 W fluorescent tubes (T4, Eurolux, Sandton, South Africa) that were placed obliquely to the fly cultures and were turned on before and after the main day lighting.

Cultures of *B. dorsalis* and C. *cosyra* were established from wild pupae provided by Citrus Research International (Nelspruit, South Africa), from which wild females were mated with laboratory-adapted males. Due to the large number of flies required for each experimental replicate, not all fly age groups or diet treatments could be tested at the same time for each species-lure combination. Additional delays in testing due to heavy rainfall also limited the capacity to simultaneously test all flies from one replicate. As a result of this, several generations of flies were needed to complete each replicate of testing, with experimental flies being taken from the fifth to the thirteenth laboratory-adapted generations. Cultures of *C. capitata* were originally established from coffee plants grown on the ARC Burgershall Research Station (25°06’39.0"S 31°05’02.0"E) near Hazyview, Mpumalanga, South Africa. All cultures were refreshed with wild flies once a year to introduce genetic variability. Pupae were placed in mesh insect cages (32.5 × 32.5 × 32.5 cm; BugDorm 43 − 030, MegaViewScience, Taichung, Taiwan, or 30 × 30 × 30 cm, Small white breeding cage, Mad Hornet Entomological Supplies, South Africa) with unrestricted access to water (water-soaked cotton wool) and food (sugar and hydrolysed yeast in a 3:1 ratio). New generations, as well as experimental flies, were obtained by allowing females of peak reproductive age (10–30 days for *Ceratitis* species and 20–40 days for *B. dorsalis* (Arita 1982; Wee and Tan (2000) to lay eggs in a 125 mL plastic container (Plastilon, South Africa). The container was covered with a layer of laboratory film (Parafilm M, Bemis, USA) that was pierced several times with an entomological pin and contained water-soaked tissue and 3 mL of guava juice concentrate (Hall’s, Tiger Consumer Brands Limited, Bryanston, South Africa) to encourage oviposition. For *B. dorsalis*, a slice of guava fruit was added to this oviposition container to further encourage oviposition. Eggs were washed out of the oviposition container with distilled water and were placed on carrot-based larval rearing medium (Citrus Research International, Nelspruit, South Africa) at a density of approximately 3 eggs/mL of medium. The container of inoculated larval medium was placed in a 2 L plastic box with a ventilated lid and a layer of sand, which was kept in the climate room, under the same conditions that fly cultures were maintained. After 15 days (within the pupal phase) the sand was sifted, and pupae were placed in a Petri-dish (ø 65 mm) and transferred into a mesh insect cage (32.5 × 32.5 × 32.5 cm, or 30 × 30 × 30 cm).

Experimental flies were produced in the same manner, except for groups with different diet treatment groups. Flies that were deprived of protein in their adult diet had unrestricted access to sugar and water but were not provided with hydrolysed yeast. Experimental flies were kept in mesh insect cages (32.5 × 32.5 × 32.5 cm, or 30 × 30 × 30 cm) until 24 h prior to semi-field testing. Flies were then transferred to transparent plastic 1 L cage with a ventilated lid and unrestricted access to water and their diet treatment specific food. With the exception of the time when they were transferred between cages, experimental flies were kept in the fly culture climate room (as detailed above).

### Correlates of Fly Responses to Commercially Used Lures

Semi-field testing was conducted in hexagonal field cages on the University of Pretoria Innovation Africa Campus, Hatfield, South Africa. Each field cage was constructed from white 50% shade cloth with a PVC floor, and was approximately 2.3 m tall and 3 m wide (at the widest points). White shade cloth was selected for the field cages to minimise flies settling on the walls (Collins et al. 2008). Three potted citrus trees (a combination of Midknight Valencia Orange and Star Ruby Grapefruit) were placed in each field cage as a habitat for flies to rest and feed during the day.

Lures that were tested for *C. capitata* were three-component BioLure Fruit Fly (Chempac), Trimedlure (TML polymeric plug, Chempac) and E.G.O PheroLure (Insect Science (Pty.) Ltd, South Africa). *Ceratitis cosyra* was tested using the same lures as *C. capitata*, except for Trimedlure, as *C. cosyra* does not respond to this lure. Lures that were tested for *B. dorsalis* were BioLure and methyl eugenol (Chempac ME polymeric plug, Chempac). All tested lures are sold commercially for fruit fly trapping. BioLure is a food-based synthetic attractant, composed of ammonium acetate, trimethylamine hydrochloride, and putrescine (Epsky et al. 1998). E.G.O PheroLure is a male attractant consisting of enriched ginger root oil and multiple sesquiterpenes, such as the sesquiterpene hydrocarbon α-copaene (Mwatawala et al. 2013; Shelly et al. 2002). Trimedlure is also a male attractant consisting of tert-butyl esters of 4- and 5-chloro-2-methylcyclohexanecarboxylic acids (Beroza et al. 1961). The last male lure tested was methyl eugenol, a phenylpropanoid that is found in many plants and is derived from eugenol (a phenylalanine product) (Tan and Nishida 2012). One lure was placed in a yellow bucket trap (Chempac) containing an insecticide block (Vapona Ag-strip, Acorn Group, South Africa) and placed in foliage in each field cage. Another unbaited trap with insecticide (control trap) was also placed in each field cage. A control trap is important when recapturing flies within a restricted space to account for random entry of flies into traps and possible visual attraction of flies to the yellow colour of the bucket traps.

For each species and lure combination, pupae were divided into three batches and dyed with three contrasting fluorescent pigment colours (T-series, Swada, UK), at a concentration of 2 g/L of pupae (Makumbe et al. 2017). Four colours were used to distinguish between the three age groups, and colours were rotated at random between each replicate. These colours were Astral Pink 1, Blaze 5, Stellar Green 8, Lunar Yellow 27, and Comet Blue 60. Each cage contained pupae of the same age, which were thus dyed the same colour. Colours were rotated amongst the age groups throughout the different replicates. Cages were furnished with sugar, or sugar and hydrolysed yeast, plus a source of water, to represent protein-deprived and protein-rich adult diets, respectively. Fly responses to each lure were determined in relation to their sex, diet, and age. For the two *Ceratitis* species, at ages two-, 10-, and 20-days after adult emergence, 25 females and males of each diet treatment were released at four times during the day (06:30, 10:00, 13:30, 17:00, UTC (+ 02:00) and left for 90 min to move around the field cage and respond to the lure. For *B. dorsalis*, the youngest cohort of flies was released at 4 days of age when still sexually immature (Wee and Tan 2000). The three age groups represent different developmental stages (Arita 1982; Wee and Tan 2000). Flies were given up to two hours to acclimatise to field conditions before the testing period began. After each 90 min period, traps were emptied, and unresponsive flies were caught with an aspirator. Responsive and unresponsive flies were transferred to separate 1.5 mL microcentrifuge tubes and stored in a freezer at -80 ˚C. Each field cage had its own separate microcentrifuge tubes for each 90 min testing period. Responsive flies were classified as individuals that were caught in the baited trap and those that did not respond to any trap, or were caught in the control trap, were classified as unresponsive flies. During each 90 min period, minimum, maximum, and average temperature, and relative humidity were recorded within the shade house using a data logger (DS1923L-F5, 1-Wire Hygrochron, iButtonLink Technology, USA). Data loggers were placed in Stevenson screens, which were placed in the foliage next to the baited yellow bucket trap. Stevenson screens were 3D-printed using white polyactic acid (PLA) filament (Vagish 2023). They consisted of a stacked tower of interlocking cone-shaped parts, with ventilation holes in the middle sections to allow for air circulation through the parts. Furthermore, light intensity was recorded at the base of the trap at the beginning and end of each 90 min period using a light meter (Model 407,026, Extech Instruments, USA).

The response of each species to the lure was calculated for each sex, diet, age, and time of day combination by subtracting the number of flies caught in the control trap (unbaited trap) from the number of flies in the experimental trap (baited trap). Only males were tested in their response to methyl eugenol, as females do not respond to this lure. For each species, age, diet and lure combination, this procedure was replicated four times. Due to fly availability, different experimental cohorts were not tested at the same time. All semi-field testing was conducted in the warmer months between late spring and early autumn (September to April) to avoid winter temperatures that would limit flights (cite Makumbe et al. 2020) and potentially trap captures.

### Nutritional Body Composition Assays

A subsample of five responsive and unresponsive flies randomly selected from each experimental variable combination of the semi-field lure response tests was used to establish the role of nutrient stores on responsiveness to methyl eugenol. Flies were thawed to room temperature (~ 20 °C) and weighed on a microbalance (to 0.001 mg, CPA2P, Sartorius AG, Germany) to determine their wet weight. Flies were then freeze-dried and weighed (to 0.001 mg) again to determine their dry weight. Water content of each fly was calculated by subtracting the dry weight from the wet weight.

To estimate the carbohydrate, lipid and protein content from the same individual, we used the methods described by Foray et al. (2012) that have been adapted for tephritid analysis (Weldon et al. 2019). All assays were colourimetric, with estimates of the component of interest determined by reading sample absorbance against a standard curve with a microplate reader (Eon Microplate Spectrophotometer, Biotek Instruments, Winooski, VT, USA). Each fly was individually homogenised in 180 µL of phosphate-buffered saline (PBS) (1.35 mM potassium chloride, 68.5 mM sodium chloride, pH 7.4) (P4417, Sigma Aldrich, Saint-Louis, MO, USA) using a microtube homogeniser (BeadBug™ 3 Position Bead Homogeniser, Benchmark Scientific, Sayreville, NJ, USA) and a 3 mm Ø zirconium bead. Flies were homogenised at 4000 rpm for one minute. The samples were then centrifuged at 200 RCF at 4 °C for 15 min.

To determine estimates of soluble protein, 1.5 µL supernatant was transferred to a 96-well microplate in duplicate. The Bradford assay (Bradford 1976) was used to determine total protein content using Bradford reagent (Sigma-Aldrich, St Louis, MO, USA). Serial dilutions of bovine serum albumin standard (P0834-10 × 1mL, Sigma-Aldrich) were used to create a standard curve (0, 25, 50, 100, 200 µg/mL). Optical density was read at 595 nm.

For carbohydrate and lipid determinations, 20 µL 20% Na_2_SO_4_ (w/v), 4.5 µL PBS buffer, and 1500 µL chloroform: methanol (1:2 v/v) was added to the remaining homogenate. Samples were then vortexed for one minute and centrifuged at 200 RCF at 4 °C for 15 min. One hundred microlitres of the supernatant was set aside, in duplicate, in 2 mL Eppendorf tubes for lipid analysis. The remainder of the supernatant was evaporated overnight in a fume hood and then resuspended using 250 µL chloroform: methanol (1:2 v/v). The anthrone method was used to determine the water-soluble carbohydrate content of these samples, with a glucose dilution range (0, 0.1, 0.25, 0.5, 1.0, 2.5, 5.0 mg mL^− 1^) used as a calibration standard. Two hundred microlitres of standard or sample were transferred into a 15 mL tube with 4.8 mL of anthrone reagent (1.42 g/L in 70% sulphuric acid) (Anthrone, ACS reagent 97% obtained from Sigma-Aldrich). Each sample and standard was incubated at 90 °C for 15 min in a water bath and then cooled on ice for five minutes. Two hundred microlitres of each sample and standard was transferred into a 96-well microplate, in duplicate, and the absorbance was read at 625 nm.

Total lipid determinations were made using the vanillin colourimetric assay. Calibration standards were made by dissolving glyceryl trioleate (Sigma-Aldrich) in chloroform: methanol (1:2 v/v) (dilution range 0, 0.1, 0.2, 0.5, 1.0 µg µL^− 1^). Samples were completely evaporated under a fume hood overnight. After which samples were incubated with 10 µL 98% sulphuric acid at 90 °C for two minutes and then cooled on ice for five minutes. Then, 210 µL of vanillin reagent (1.2 g L^− 1^ vanillin dissolved in 68% orthophosphoric acid) was added to the samples (Vanillin, ReagentPlus®, 99% obtained from Sigma-Aldrich). The microplate was shaken for 15 min at room temperature and the absorbance was read at 525 nm and compared to the calibration standards.

### Lure Formulation Weight Change

To evaluate the effects of temperature and relative humidity on the change in weight of lure formulations, we purchased the same commercial formulations of BioLure, methyl eugenol, and E.G.O PheroLure from suppliers and stored them until use in a refrigerator (~ 6˚C). For Trimedlure, we used Capilure capsules (River Bioscience, Gqeberha, South Africa) and stored them at ~-20˚C following manufacturer instructions. Capilure was used in this experiment because it is recommended for monitoring of *C. capitata* in the South African citrus industry. Each lure type was weighed and placed in a separate yellow bucket trap suspended in a mixed citrus orchard on the University of Pretoria Innovation Africa Campus. One of each lure type was located at the northern edge, centre and southern edge of the lemon orchard. Temperature and relative humidity at each location within the lemon orchard was recorded using temperature and relative humidity data loggers placed within Stevenson screens hung in the foliage. At daily intervals over a period of 30 days, weight of each lure was recorded to determine weight loss in relation to daily mean temperature and relative humidity. The experiment was replicated five times to encompass differences in temperature and natural relative humidity (Table 1).

**Table 1:** Weekly mean, maximum and minimum temperatures and relative humidity recorded in a mixed citrus orchard during assessment of lure formulation loss in the field for a duration of 30 days. The orchard was located on the University of Pretoria Innovation Africa Campus, Hatfield, South Africa. Trimedlure (Capilure) was tested in the four periods in 2020. BioLure, E.G.O PheroLure and methyl eugenol were tested during the five periods in 2021

Environmental conditions	2020					2021			
	25/06	29/07	01/09	06/10	19/11	15/01	16/02	23/03	10/05
Average (± s.e.) weekly temperature (°C)	11.75 (0.16)	14.49 (0.15)	19.13 (0.14)	21.89 (0.15)	20.95 (0.10)	21.80 (0.09)	21.42 (0.12)	18.69 (0.13)	13.52 (0.15)
Maximum temperature (°C)	31.09	41.57	32.65	45.11	35.15	41.57	37.64	39.14	38.14
Minimum temperature (°C)	-3.48	0.59	6.12	10.08	12.08	14.08	11.64	6.56	0.04
Average (± s.e.) weekly relative humidity (%)	48.92 (0.52)	45.48 (0.49)	48.73 (0.52)	58.30 (0.54)	77.89 (0.42)	79.22 (0.45)	66.49 (0.49)	69.82 (0.51)	57.85 (0.50)
Maximum relative humidity (%)	100	100	100	100	100	100	100	100	99.22
Minimum relative humidity (%)	3.82	4.03	5.54	11.46	24.58	9.89	11.55	11.84	9.17

### Data Analysis

All statistical analyses were performed using R software (v 4.1.0, The R Foundation for Statistical Computing). Data were analysed with generalized linear mixed effects models by using the “glmer” and “lmer” procedures from the lme4 package (Bates et al. 2015). Replicate was included as a random effect for all models. Due to known differences between species in the lures to which flies are attracted and their specificity to the sexes (Tan et al. 2014), each species was analysed separately.

The response variable for intrinsic and extrinsic factor models was the number of flies caught in the baited trap minus the flies caught in the control trap. When the number of flies in the control trap exceeded that of the baited trap, trap catch was set to zero flies (this was done 144 times out of 1344 tests). A test comprised of measuring the response of one combination of age, sex, and diet for a specific species-lure combination during a 90 min period. Fixed effects for models testing intrinsic factors were adult diet, fly age, and sex. For each species-lure combination only experimental groups with the strongest positive response to the lure were used to assess the effect of temperature, humidity, and light intensity. This was done to ensure that only flies that were motivated to respond to the lure were used to assess how environmental variables affect the number of flies caught by a baited trap. Furthermore, this approach enabled us to fit the data to standard data distributions during analysis. Time of day was excluded from these analyses as it conflated with temperature, relative humidity and light intensity (Fig. S1). Similar to the approach used by Bunning et al. (2016), sequential model building was used to assess the linear and non-linear effects of extrinsic factors on fly response. First, a regression model was run to evaluate how the number of flies caught in baited traps was affected by the linear effects of temperature (°C), relative humidity (% RH), light intensity (lux). Then, the non-linear, quadratic effects of temperature × temperature, humidity × humidity, and light intensity × light intensity on flies caught in baited traps were tested in a multivariate non-linear regression model including the linear and non-linear effects. Akaike’s information criterion was used to compare linear and multivariate non-linear regression models to assess whether the multivariate non-linear effects of extrinsic factors were part of the minimum adequate model. Gaussian distributions were used for models testing extrinsic and intrinsic factors, while the transformations of response variables varied between models (Supplementary material). Response variables were transformed due to their non-normal distribution and the non-constant variation of residuals. Overall, transforming the response variables homogenised the variance and increased model fit.

Models testing the effect of body nutrient composition on fly responsiveness to lures used a binomial family and differentiated between flies that were caught in the baited trap (responsive) versus flies that were not caught in the baited trap (non-responsive). The purpose of this analysis was to define the carbohydrate, lipid and protein levels driving lure responsiveness rather than to compare levels of each macronutrient in different treatments. Responsive flies were given a score of 1, and non-responsive flies were given a score of 0. Fixed effects for models for responsiveness relative to body composition were weight, protein content, lipid content, carbohydrate content, adult diet, age and sex.

Analysis of variance tables were generated using type III sums of squares to summarise the effect of factors in the minimum adequate models resulting from the procedures described above. If no interaction term was retained in the minimum adequate model, tables were generated using type II sums of squares. If a significant interaction effect was found, post hoc pairwise comparison tests were performed using estimated marginal means from the ‘emmeans’ function and the package of the same name (Russel, 2020). Coefficients were taken from the function summary table. Post hoc pairwise comparisons were not needed for significant main effects because no more than two levels comprised each factor.

Weekly weight change from lures was related to weekly average temperature and relative humidity using linear mixed effects models. This was done using the ‘lmer’ function from the lme4 package (Bates et al. 2015). The random effects of lure dispenser identity and ‘month’ were included in the model. Three-dimensional plots were created to display the contribution of temperature and relative humidity to lure weight loss.

## Results

### *Ceratitis* *capitata*

#### Biolure

**Fig. 1:**
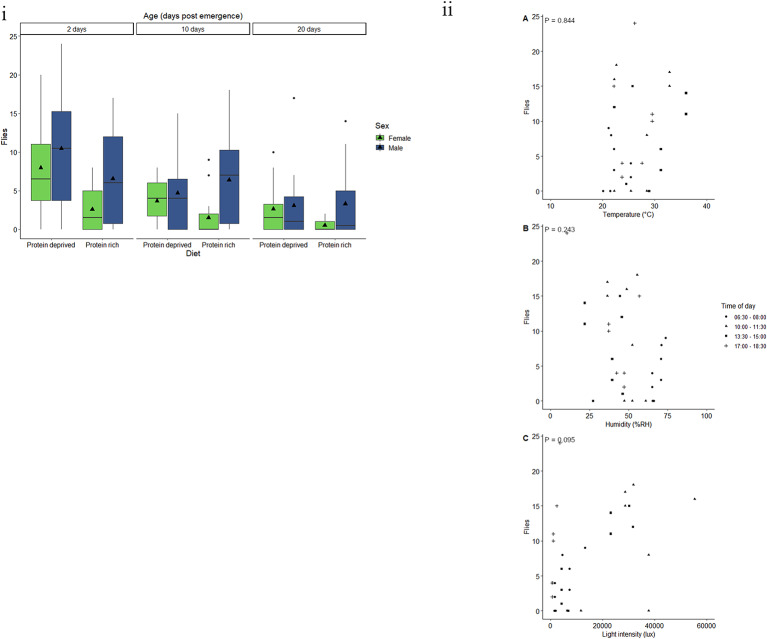
Response by *C. capitata*to BioLure varies according to physiological and environmental factors. (i) Trap captures as a function of age for male (green) and female (blue) *C. capitata* fed a protein-rich or protein-deprived diet. Black triangles represent the mean corrected trap catch per experimental group. Each group represent 25 flies and was given 90 min in a semi-field cage to respond to a Biolure baited yellow bucket trap. Black triangles represent the mean trap catch per experimental group. (ii) Trap captures of the most responsive experimental groups of *C. capitata* (two- or 10-day-old males fed a protein-deprived diet) at different (**A**) temperatures (°C), (**B**) relative humidity (% RH), and (**C**) light intensities (lux). Trendlines, 95% confidence interval bands, and the equation of the relationship between factors are only shown when an environmental variable had a significant effect (*P* < 0.05) on trap capture

Age, diet and the interaction between diet and sex had a significant effect on the response of *C. capitata* to BioLure (Table 2). Twenty-day-old male and female had the lowest response to BioLure with a mean of 0.3 flies being caught (Fig. 1i). There was a 325.5% and 440.9% increase in fly response in 10- and 2-day-old flies respectively, when compared with 20-day-old flies. Furthermore, an average of 5.4 flies reared on a protein-deprived diet were caught using Biolure, whereas the response of flies fed on a protein-rich diet was 35.8% lower. Additionally, females fed a protein-deprived diet had a higher response than females that received a protein-rich diet (*t* = 4.152, P = < 0.001). However, there was no significant difference between diet treatments for males (*t* = 1.018, *P* = 0.31).

Only protein content significantly affected the likelihood of response to BioLure by *C. capitata* (Table 2), with flies having higher protein content being more responsive to the bait. Across all ages, the protein content at which there is a 50% probability of response (R_50_) was 712.5 µg (Fig. S2a).

Male that were 2- or 10-days-old and were fed a protein-deprived diet were used to assess the effect of abiotic variables. The minimum adequate model included the linear effects of temperature, humidity, and light intensity. However, neither temperature, humidity nor light intensity had a significant effect on the response of *C. capitata* males to BioLure (Table 3, Fig. 1ii).

#### E.G.O PheroLure

**Fig. 2:**
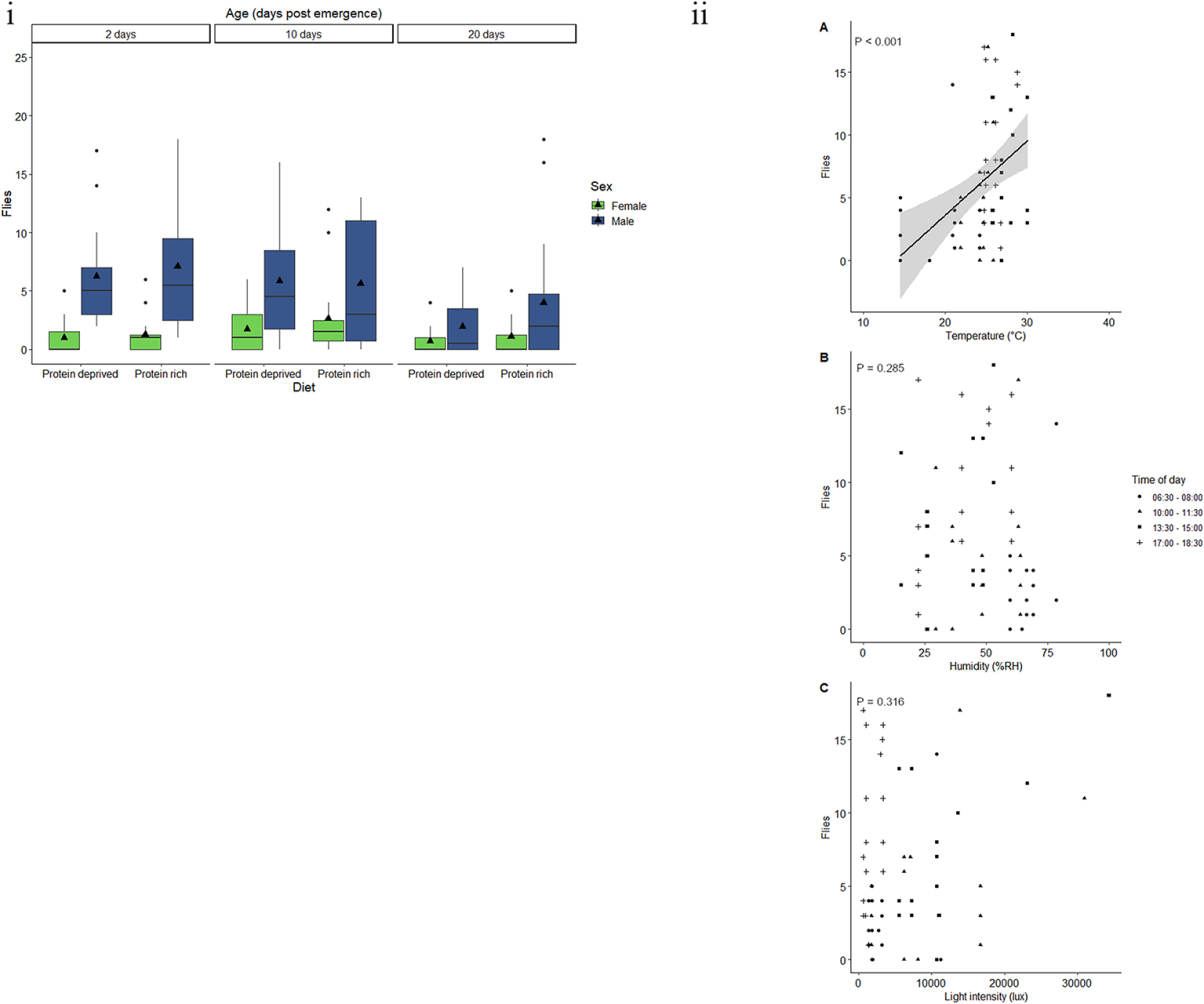
Response by *C. capitata*to E.G.O PheroLure varies according to physiological and environmental factors. (i) Trap captures as a function of age for male (green) and female (blue) *C. capitata* fed a protein-rich or protein-deprived diet. Each group represent 25 flies and was given 90 min in a semi-field cage to respond to a E.G.O PheroLure baited yellow bucket trap. Black triangles represent the mean trap catch per experimental group. (ii) Trap captures of the most responsive experimental groups of *C. capitata* (two- or 10-day-old males fed either protein-deprived, or protein-rich diets) at different (**A**) temperatures (°C), (**B**) relative humidity (%RH), and (**C**) light intensities (lux). Trendlines, 95% confidence interval bands, and the equation of the relationship between factors are only shown when an environmental variable had a significant effect (*P* < 0.05) on trap capture

Sex and the interaction between age and sex had a significant effect on the response to traps baited with E.G.O PheroLure (Table 2). On average, male *C. capitata* responded to E.G.O PheroLure 265.2% more than females (Fig. 2i). There was no significant difference between 2- and 10-day-old males (*t* = -1.718, *P* = 0.202), with an average of 6.7 and 5.8 males caught, respectively. An average of 3.0 20-day-old males were caught. This is a significant decrease of 48.4% from 10-day-old males (*t* = 3.249, *P* = 0.004) and 55.6% from 2-day-old males (*t* = 4.967, *P* < 0.001).

Neither age, diet, weight, protein content, lipid content, nor carbohydrate content had a significant effect on the likelihood of *C. capitata* to respond to E.G.O PheroLure (Table 2, Fig. S3a, b, c, d).

Two- or 10-day-old males fed either protein-deprived, or protein-rich diets were used to assess the effect of temperature, humidity, and light intensity. The minimum adequate model included the linear effects of temperature, humidity, and light intensity. Temperature had a significant effect on the response of *C. capitata* to E.G.O PheroLure (Table 3), with response increasing with temperature (Table 3, Fig. 2ii). Model predictions estimated that flies are unlikely to to respond at temperatures below 22.5 °C, 95% CI [21.24, 23.82], and that an increase in response of 0.3 flies is predicted for every 1 °C increase in temperature. Humidity and light intensity did not have a significant effect on the response of *C. capitata* to E.G.O PheroLure baited traps (Table 3).

#### Trimedlure

**Fig. 3:**
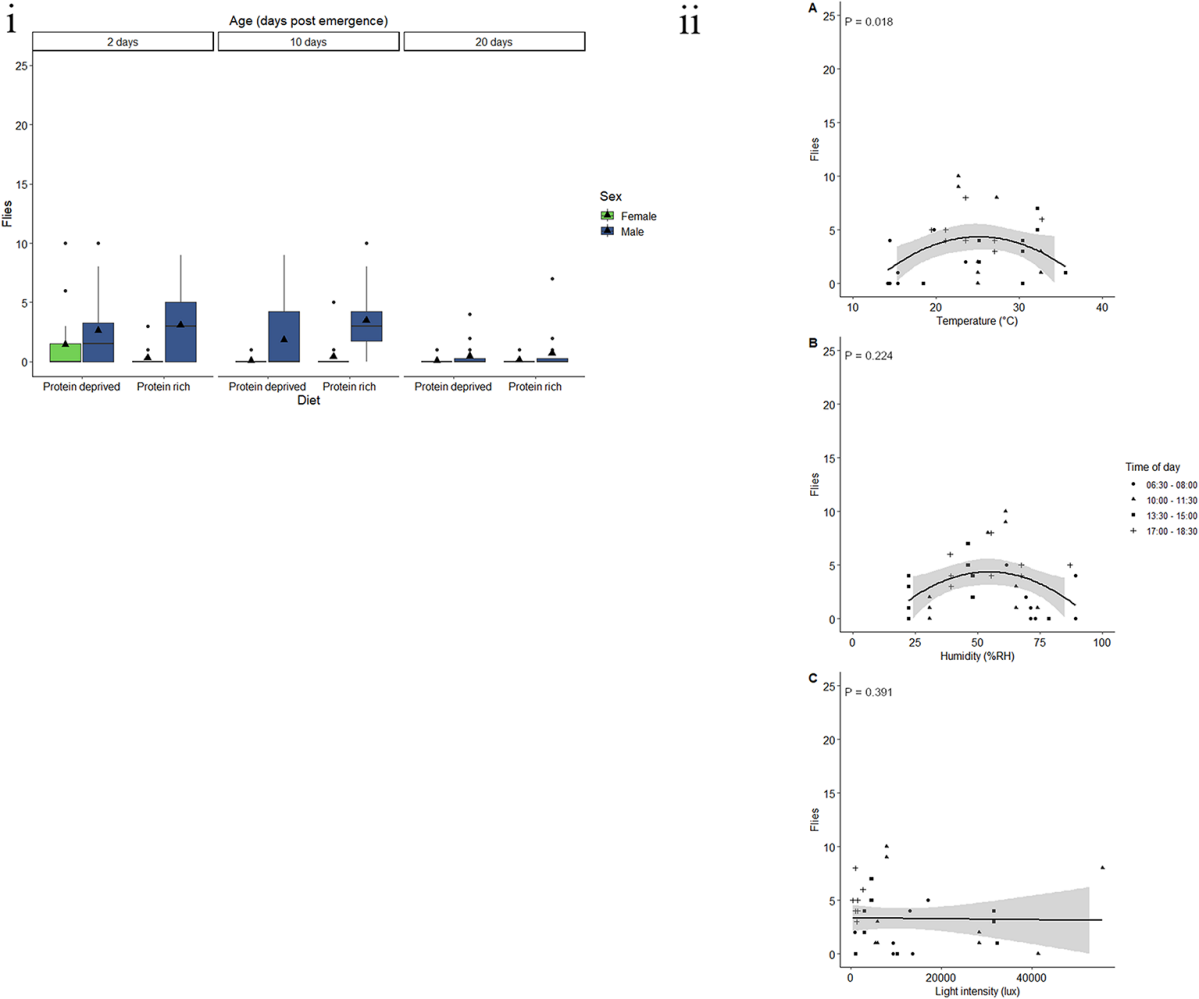
Response by *C. capitata*to trimedlure varies according to physiological and environmental factors. (i) Trap captures as a function of age for male (green) and female (blue) *C. capitata* fed a protein-rich or protein-deprived diet. Each group represent 25 flies and was given 90 min in a semi-field cage to respond to a Biolure baited yellow bucket trap. Black triangles represent the mean trap catch per experimental group. (ii) Trap captures of the most responsive experimental groups of *C. capitata* (two- or 10-day-old males fed a protein-rich diet) at different (**A**) temperatures (°C), (**B**) relative humidity (%RH), and (**C**) light intensities (lux). Trendlines, 95% confidence interval bands, and the equation of the relationship between factors are only shown when an environmental variable had a significant effect (*P* < 0.05) on trap capture

Age and sex had a significant effect on the response of *C. capitata* to Trimedlure (Table 2). The two-day-old flies were most responsive, with an average catch of 1.9 flies (Fig. 3i). In comparison to two-day-old flies, response decreased by 21.3% for 10-day-old flies and by 81.5% for 20-day-old flies, with an average of 1.5 and 0.3 flies caught, respectively. An average of 2.1 males were caught in Trimedlure baited traps. The response of females decreased by 81.1% to an average catch of 0.4 flies.

Wet weight had a significant effect on the likelihood of *C. capitata* to respond to Trimedlure (Table 2), with response increasing in heavier flies (Fig. S4a). A 50% probability of response (R_50_) for *C. capitata* to respond to Trimedlure occurs when a fly has an average weight of 6.47 mg. Protein content also had a significant effect (Table 2), with the likelihood of fly response decreasing with higher protein levels. A 50% probability (R_50_) of response to Trimedlure occurs at 724.64 µg of protein.

Only experimental groups with the strongest positive response to Trimedlure were used to assess the effect of temperature, humidity, light intensity, and time of day. Males that were two- or 10-days-old and fed a protein-rich diet were used to assess the effect on abiotic variables on fly response to Trimedlure. The minimum adequate model included the linear and the non-linear quadratic effects of temperature, humidity, and light intensity. Neither the linear, nor the non-linear effects of humidity or light intensity had a significant effect on fly response (Table 3). The linear and non-linear quadratic effects of temperature had a significant effect on fly response (Table 3). The quadratic effects of temperature had a positive effect on response to Trimedlure, with the effects of temperature becoming stronger as temperature changes (Table 3). From the model, *C. capitata* was predicted to be caught in Trimedlure baited traps at temperatures between 12.21 °C, 95% CI [11.28, 13.14] and 37.79 °C, 95% CI [36.86, 38.72] (Fig. 3ii). Peak response occurred at 25.00 °C, with an average of 4.3 flies.

**Table 2:** The effects of intrinsic and nutritional factors on the response of *C. capitata* to three attractants

Attractant		χ2	df	*P*
Biolure				
Intrinsic	Diet	**6.789**	**1**	**0.009**
	Age	**9.081**	**2**	**0.011**
	Sex	0.073	1	0.787
	Diet × Age	0.912	2	0.634
	Diet × Sex	**5.334**	**1**	**0.021**
	Age × Sex	0.662	2	0.718
	Diet × Age × Sex	1.591	2	0.451
Nutritional	Wet weight	1.565	1	0.211
	Protein	**11.115**	**1**	**< 0.001**
	Lipid	1.381	1	0.240
	Carbohydrate	0.095	1	0.758
	Age	0.496	1	0.780
	Sex	0.030	1	0.862
	Diet	0.181	1	0.671
E.G.O Pherolure				
Intrinsic	Diet	0.312	1	0.576
	Age	3.843	2	0.146
	Sex	**6.252**	**1**	**0.012**
	Diet × Age	0.126	2	0.939
	Diet × Sex	0.650	1	0.420
	Age × Sex	**7.427**	**2**	**0.024**
	Diet × Age × Sex	0.812	2	0.666
Nutritional	Wet weight	0.045	1	0.832
	Protein	2.669	1	0.102
	Lipid	0.071	1	0.790
	Carbohydrate	0.022	1	0.881
	Age	2.243	2	0.326
	Diet	2.104	1	0.147
Trimedlure				
Intrinsic	Diet	2.948	1	0.086
	**Age**	**23.42**	**2**	**< 0.001**
	**Sex**	**50.632**	**1**	**< 0.001**
Nutritional	**Wet weight**	**5.332**	**1**	**0.021**
	**Protein**	**10.942**	**1**	**< 0.001**
	Lipid	561	1	0.454
	Carbohydrate	3.779	1	0.052
	Age	3.391	2	0.183
	Diet	0.549	1	0.459

*Note* Bold values indicate significant terms.

**Table 3:** The effects of extrinsic factors on the response of *C. capitata* to three attractants

Attractant		Coefficient ± SE	χ2	df	*P*
BioLure					
Linear effects	Temperature	-0.011 ± 0.057	0.039	1	0.844
	Humidity	-0.018 ± 0.015	1.360	1	0.243
	Light intensity	0.000 ± 0.000	2.783	1	0.095
E.G.O Pherolure					
Linear effects	Temperature	**0.090 ± 0.024**	**13.420**	**1**	**< 0.001**
	Humidity	0.006 ± 0.005	1.145	1	0.285
	Light intensity	0.000 ± 0.000	0.905	1	0.316
Trimedlure					
Linear effects	Temperature	**0.443 ± 0.170**	**6.781**	**1**	**0.009**
	Humidity	0.047 ± 0.038	1.48	1	0.224
	Light intensity	0.000 ± 0.000	0.736	1	0.391
Non-linear effects	Temperature × Temperature	**-0.008 ± 0.003**	**5.626**	**1**	**0.018**
	Humidity × Humidity	-0.000 ± 0.000	0.637	1	0.425
	Light intensity × Light intensity	-0.000 ± 0.000	0.597	1	0.440

*Note* Bold values indicate significant terms

### *Ceratitis* *cosyra*

#### BioLure

**Fig. 4:**
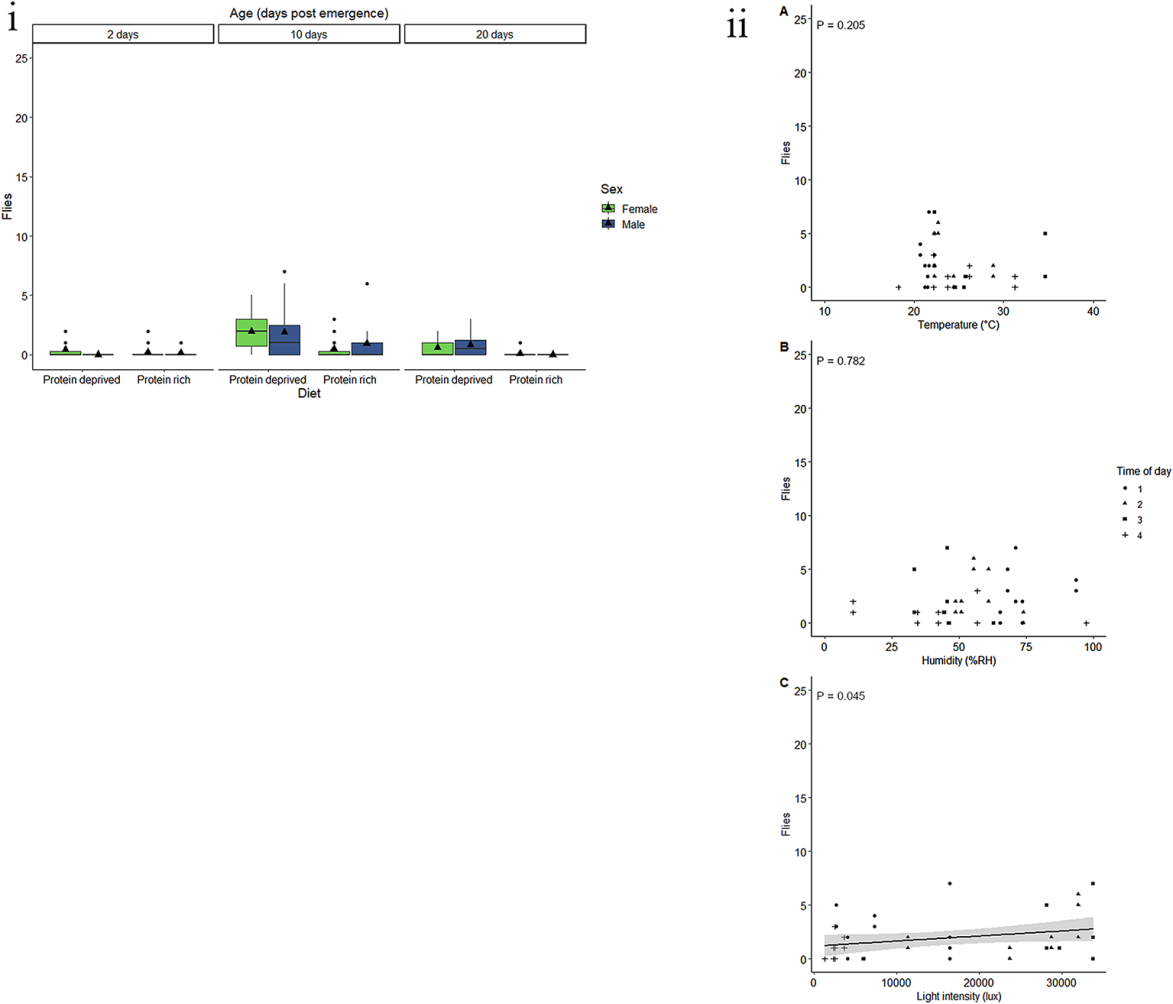
Response by *C. cosyra*to BioLure varies according to physiological and environmental factors. (i) Trap captures as a function of age for male (green) and female (blue) *C. cosyra* fed a protein-rich or protein-deprived diet. Each group represent 25 flies and was given 90 min in a semi-field cage to respond to a Biolure baited yellow bucket trap. Black triangles represent the mean trap catch per experimental group. (ii) Trap captures of the most responsive experimental groups of *C. cosyra* (two- or 10-day-old males and females fed a protein-deprived diet) at different (**A**) temperatures (°C), (**B**) relative humidity (%RH), and (**C**) light intensities (lux). Trendlines, 95% confidence interval bands, and the equation of the relationship between factors are only shown when an environmental variable had a significant effect (*P* < 0.05) on trap capture

Diet and age had a significant effect on the response of *C. cosyra* to BioLure (Table 4). An average of 1.05 flies fed a protein-deprived diet across all age groups were caught in BioLure baited traps (Fig. 4i). There was a 65.4% decrease in response in flies fed a protein-rich diet, with an average of 0.4 flies. The highest response to BioLure occurred at 10-days-old, with an average of 1.33 flies being caught. In contrast to 10-day-old flies, there was a 71.7% decrease in response in 20-day-old and an 85.9% decrease in response in two-day-old *C. cosyra*, with an average of 0.4 and 0.2 flies caught, respectively.

Neither weight, protein content, carbohydrate content, diet, or sex had a significant effect on the likelihood of *C. cosyra* to respond to BioLure (Table 4, Fig. S5a, b, c, d. Lipid content and fly age did have a significant effect (Table 4), with response decreasing with lipid content. A 50% probability (R_50_) of response occurs at 500 µg of lipid. The effect of age was similar to that described above, with a higher response seen in 10-day-old flies.

Males and females that were 10-, or 20-days-old and were fed a protein-deprived diet were used to assess the effect on abiotic variables on fly response to Biolure. The minimum adequate model included the linear effects of temperature, humidity, and light intensity. Only the linear effect of light intensity had a significant effect on lure response, with response increasing with light intensity (Table 5). The model predicted that no flies would respond to BioLure at light intensities below 12,578 lx, 95% CI [12577.34, 12578.66], and that there was an increase in response of 0.5 flies for every 10,000 lx increase in light intensity (Fig. 4ii).

#### E.G.O PheroLure

**Fig. 5:**
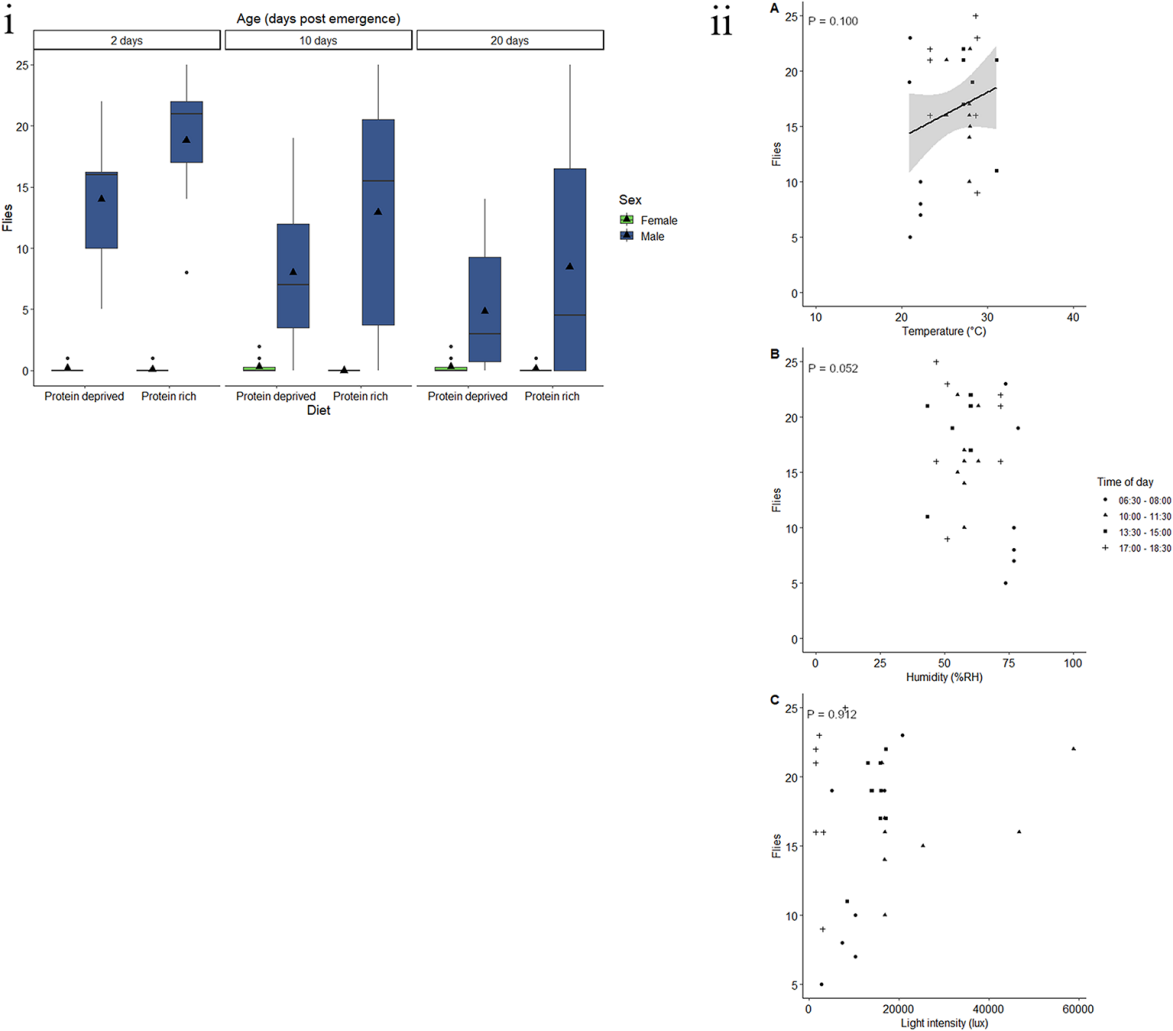
Response by ***C. cosyra***to E.G.O PheroLure varies according to physiological and environmental factors. (i) Trap captures as a function of age for male (green) and female (blue) *C. cosyra* fed a protein-rich or protein-deprived diet. Each group represent 25 flies and was given 90 min in a semi-field cage to respond to a E.G.O PheroLure baited yellow bucket trap. Black triangles represent the mean trap catch per experimental group. (ii) Trap captures of the most responsive experimental groups of *C. cosyra* (two- or 10-day-old males fed either protein-rich, or protein-deprived diets) at different (**A**) temperatures (°C), (**B**) relative humidity (%RH), and (**C**) light intensities (lux). Trendlines, 95% confidence interval bands, and the equation of the relationship between factors are only shown when an environmental variable had a significant effect (*P* < 0.05) on trap capture

Sex, and the interaction between age and sex had a significant effect on the response of *C. cosyra* to E.G.O PheroLure (Table 4). On average, 11.2 males were caught in E.G.O PheroLure baited traps (Fig. 5i). This response decreased by 98.5% in females, for which an average of 0.2 flies were caught. There was a significant decrease in the response from two- to 10-day-old males (*t* = -3.896, *P* < 0.001), from two- to 20-day-old males (*t* = 7.234, *P* < 0.001), and from 10- to 20-day-old males (*t* = 3.338, *P* = 0.003). However, there was no significant difference between ages for females.

Weight, protein, lipid, and carbohydrate content had no effect on the response of *C. cosyra* to E.G.O PheroLure (Table 4 Fig. S6a, b, c, d). Additionally, fly diet and age had no significant effect in this analysis (Table 4).

Males that were two- or 10-days-old and were fed either protein-rich, or protein-deprived diets were used to assess the effects of abiotic variables on fly response. The minimum adequate model included the linear effects of temperature, humidity, and light intensity. Temperature, humidity, and light intensity had no significant effect on the response of *C. cosyra* to E.G.O PheroLure (Table 5, Fig. 5ii). However, there was a significant interaction effect between temperature and relative humidity. Taken together, the response of *C. cosyra* to E.G.O. PheroLure was lowest at high humidity, low temperature conditions and peaked at mild humidity, mild temperature conditions (Fig. S7).

**Table 4:** The effects of intrinsic and nutritional factors on the response of *C. cosyra* to two attractants

Attractant		χ2		df		*P*
BioLure						
Intrinsic	Diet	**20.979**		**1**		**<0.001**
	Age	**50.148**		**2**		**< 0.001**
	Sex	0.246		1		0.620
Nutritional	Wet weight	0.464		1		0.500
	Protein	3.619		1		0.057
	Lipid	**10.042**		**1**		**0.002**
	Carbohydrate			1		0.941
	Age	**9.785**		**1**		**0.008**
	Sex	3.782		1		0.052
	Diet	0.002		1		0.964
E.G.O PheroLure						
Intrinsic	Diet	1.267		1		0.260
	Age	0.148		2		0.929
	Sex	**42.021**		**1**		**< 0.001**
	Diet × Age	0.213		2		0.899
	Diet × Sex	1.243		1		0.265
	Age × Sex	**11.635**		**2**		**0.003**
	Diet × Age × Sex	0.477		2		0.788
Nutritional	Wet weight	0.045		1		0.832
	Protein	2.669		1		0.102
	Lipid	0.071		1		0.790
	Carbohydrate	0.022		1		0.881
	Age	2.243		2		0.326
	Diet	2.104		1		0.147

*Note* Bold values indicate significant terms

**Table 5:** The effects of extrinsic factors on the response of *C. cosyra* to two attractants

Attractant		Coefficient ± SE	χ2	df	*p*
BioLure					
Linear effects	Temperature	-0.066 ± 0.052	1.609	1	0.205
	Humidity	-0.003 ± 0.009	0.077	1	0.782
	Light intensity	**0.000 ± 0.000**	**4.012**	**1**	**0.045**
E.G.O PheroLure					
Linear effects	Temperature	-0.549 ± 0.334	2.709	1	0.100
	Humidity	-0.245 ± 0.126	3.769	1	0.052
	Light intensity	-0.000 ± 0.001	0.012	1	0.912
	Temperature × Humidity	**0.010 ± 0.004**	**5.905**	**1**	**0.015**
	Temperature × Light intensity	0.000 ± 0.000	0.519	1	0.472
	Humidity × Light intensity	0.000 ± 0.000	0.953	1	0.329
	Temperature × Humidity × Light intensity	-0.000 ± 0.000	3.555	1	0.059

*Note* Bold values indicate significant terms

### *Bactrocera* *dorsalis*

#### BioLure

**Fig. 6:**
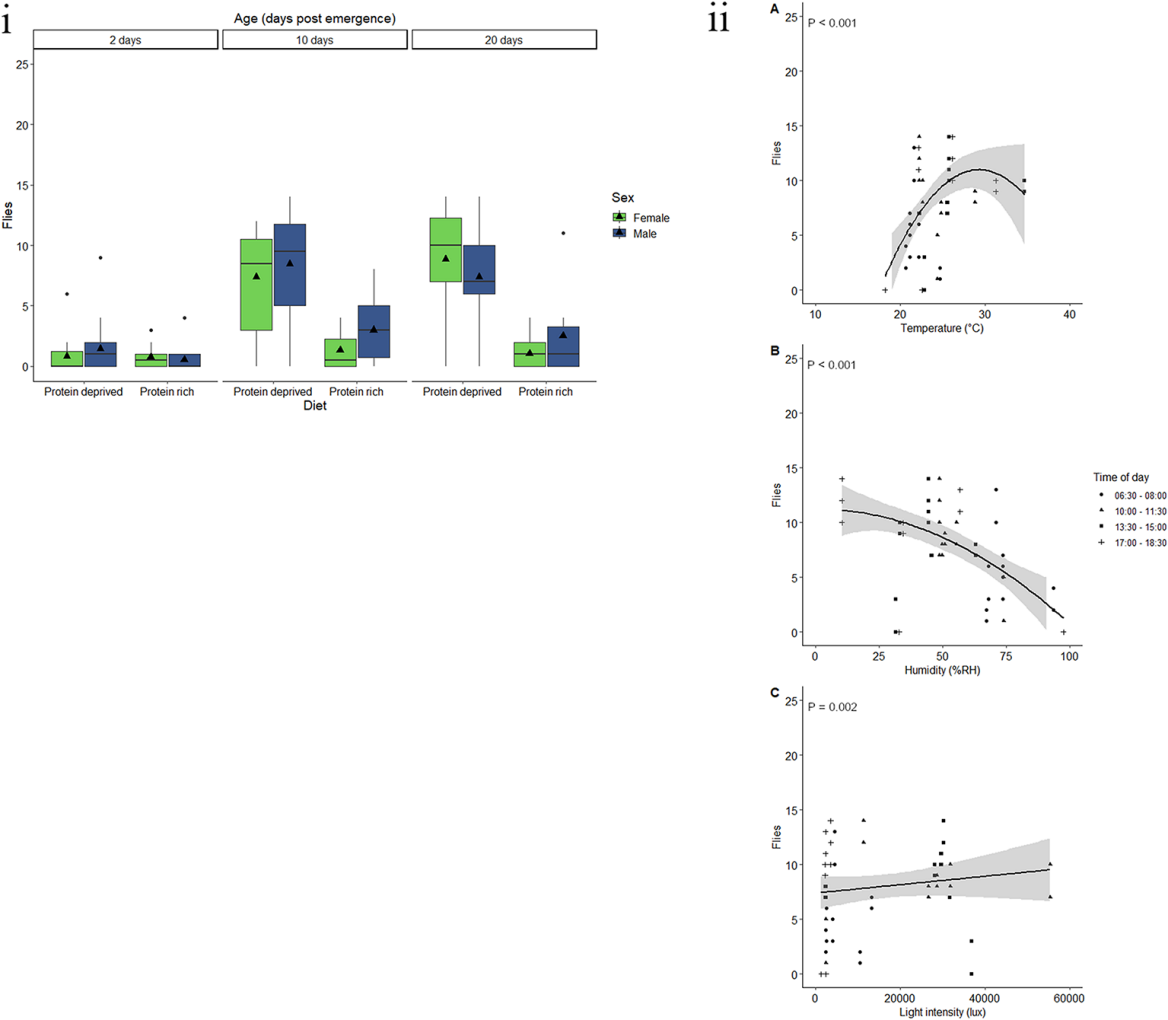
Response by *B. dorsalis*to BioLure varies according to physiological and environmental factors. (i) Trap captures as a function of age for male (green) and female (blue) *B. dorsalis* fed a protein-rich or protein-deprived diet. Each group represent 25 flies and was given 90 min in a semi-field cage to respond to a Biolure baited yellow bucket trap. Black triangles represent the mean trap catch per experimental group. (ii) Trap captures of the most responsive experimental groups of *B. dorsalis* (10-day-old males and females fed a protein-deprived diet) at different (**A**) temperatures (°C), (**B**) relative humidity (%RH), and (**C**) light intensities (lux). Trendlines, 95% confidence interval bands, and the equation of the relationship between factors are only shown when an environmental variable had a significant effect (*P* < 0.05) on trap capture

Diet, age, and the interaction between diet and age had a significant effect on the response of *B. dorsalis* to BioLure (Table 6). Across all ages, a higher response to BioLure was found in flies fed a protein-deprived diet, with an average of 5.7 flies caught (Fig. 6i). Response to BioLure decreased by 73.2% to an average of 1.53 flies being caught when flies were fed protein. The lowest response to BioLure was shown by two-day-old flies, with an average of 0.9 flies caught. The response increased by 464.9% for 10-day-old and by 456.2% for 20-day-old flies, with an average catch of 5.0 and 5.0 flies, respectively.

There was no significant difference between diets for two-day-old flies (*t* = 0.517, *P* = 0.606). However, diet significantly affected response in 10- (*t* = 6.207, *P* < 0.001) and 20-day-old (*t* = 6.759, *P* < 0.001) flies. Flies fed on a protein-rich diet had no significantly different response between two- and 20-day-old flies (*t* = -2.212, *P* = 0.072), or between 10- and 20-day-old flies (*t* = 0.622, *P* = 0.808). However, there was a significant difference between two- and 10-day-old flies that were fed a protein-rich diet (*t* = 2.834, *p* = 0.014). For flies fed a protein-deprived diet, two-day-old flies had a significantly lower response than 10- (*t* = 8.524, *P* < 0.001) and 20-day-old (t = -8.454, *P* < 0.001) flies.

Wet weight and protein content had a significant effect on the response of *B. dorsalis* to BioLure (Table 6). Heavier flies responded less to Biolure, with a 50% probability of response (R_50_) occurring when flies weigh 14.29 mg (Fig. S8a. Response increased with protein content, with a 50% probability of response occurring at 1321.43 µg of protein (Fig. S8b).

Males and females that were 10-days-old and were fed a diet deprived of protein were used to assess the effects of abiotic variables. The minimum adequate model included the linear and the non-linear, quadratic effects of temperature, humidity, and light intensity. The linear and non-linear effects of temperature and humidity had a significant effect on the response of *B. dorsalis* to BioLure (Table 7). Only the linear effects of light intensity significantly influenced fly response. Additionally, the interaction effects between temperature and humidity, temperature and light intensity, humidity and light intensity, and between temperature, humidity and light intensity had a significant effect on response. *Bactrocera dorsalis* is predicted to be caught in BioLure baited traps between 17.9 °C, 95% CI [16.81, 18.95] and 40.3 °C, 95% CI [39.27, 41.41], with the highest average response of 10.0 flies being caught at 29.1 °C (Fig. 6ii). Model predictions showed that flies should be caught at all relative humidity levels, with a peak in response predicted at 1.3% RH, 95% CI [0.23, 2.37] (Fig. 6ii). The response of *B. dorsalis* to BioLure increased with light intensity (Fig. 6ii). Model predictions estimated that the lowest response occurred at 0 lx, with 7.4 flies, 95% CI [6.33, 8.47] responding. Taken together, higher responses were found at high temperature, low humidity regions (Fig. S9). Additionally, peak responses were found at low humidity, low light intensity regions. Similarly, peak responses of *B. dorsalis* was found in low light intensity, high temperature conditions.

#### Methyl Eugenol

**Fig. 7:**
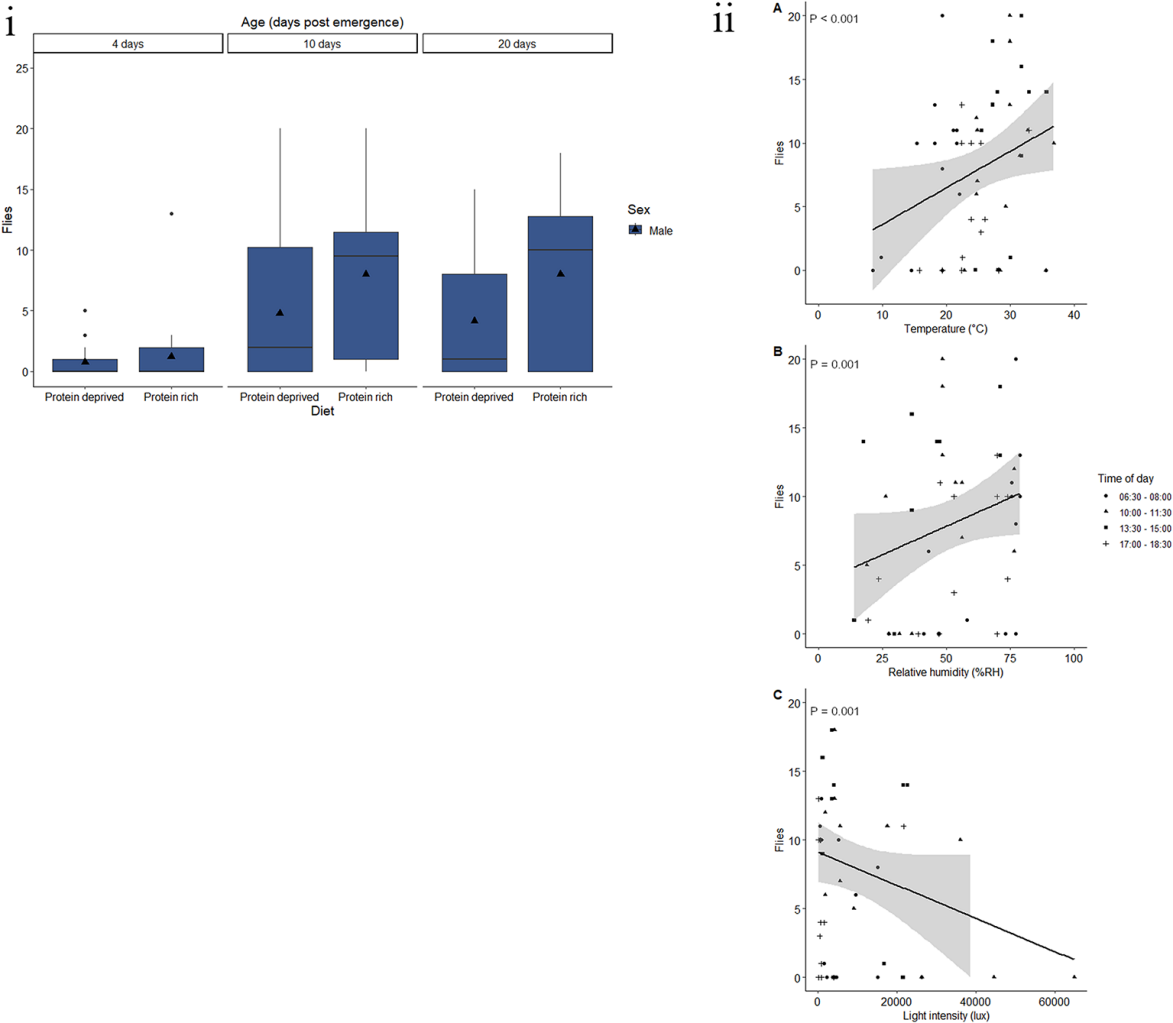
Response by *B. dorsalis*to methyl eugenol varies according to physiological and environmental factors. (i) Trap captures as a function of age for male (green) *B. dorsalis* fed a protein-rich or protein-deprived diet. Each group represent 25 flies and was given 90 min in a semi-field cage to respond to a methyl eugenol baited yellow bucket trap. Black triangles represent the mean trap catch per experimental group. (ii) Trap captures of the most responsive experimental groups of *B. dorsalis* (10- or 20-day-old males fed a protein-rich diet) at different (**A**) temperatures (°C), (**B**) relative humidity (%RH), and (**C**) light intensities (lux). Trendlines, 95% confidence interval bands, and the equation of the relationship between factors are only shown when an environmental variable had a significant effect (*P* < 0.05) on trap capture

Diet, age and the interaction between diet and age had a significant effect on the response of *B. dorsalis* to methyl eugenol (Table 6). Flies reared on a protein-rich diet had a higher response to methyl eugenol, with an average of 5.9 flies caught (Fig. 7i). Flies fed a protein-deprived diet had a 44.2% decrease in response, with an average of 3.3 flies caught per trap. Response to methyl eugenol increases with age, with four-day-old flies showing the lowest response with an average of 1.0 flies caught per trap (Fig. 7i). There was a 552.9% increase for 10-day-old flies and a 538.3% increase for 20-day-old flies, with an average catch of 6.4 and 6.3 flies, respectively.

For flies fed a diet deprived of protein, there was a significant difference between four- and 10-day-old flies (*t* = -3.679, *P* = 0.001) and four- and 20-day-old flies (*t* = -3.847, *P* < 0.001). There was no significant difference between 10- and 20-day-old flies that were fed a protein-deprived diet (*t* = 1.203, *P* = 0.454). For flies fed a protein-rich diet, there was a significant difference between 4- and 10-day-old flies (*t* = -5.353, *P* < 0.001) and between 4- and 20-day-old flies (*t* = -6.723, *p* < 0.001), but not between 10- and 20-day-old flies (*t* = -1.37, *P* = 0.36). There was no significant difference resulting from diet for four- (*t* = 0.572, *P* = 0.569) or 10-day-old (*t* = -0.933, *P* = 0.353) flies. However, there was a significant difference from the diet treatment in 20-day-old flies, with flies fed a protein-rich diet showing a higher response (*t* = -3.506, *P* < 0.001).

Neither wet weight, carbohydrate content, diet or age influenced the response of *B. dorsalis* to methyl eugenol (Table 6, Fig. S10a, c). However, protein and lipid content did have a significant effect on response (Table 6). The likelihood of response increased with higher protein content (Fig. S10b). However, the likelihood of response decreased with increasing lipid content (Fig. S10d). The amount of protein required for a 50% probability of response (R_50_) is 1222.22 µg. Whereas, a 50% probability of response (R_50_) occurs at a lipid content of 352.94 µg.

**Table 6:** The effects of intrinsic, nutritional, and extrinsic factors on the response of *B. dorsalis* to two attractants

Attractant		χ2	df	*p*
Biolure				
Intrinsic	Diet	**28.141**	**1**	**< 0.001**
	Age	**63.022**	**2**	**< 0.001**
	Sex	0.144	1	0.705
	Diet × Age	**25.604**	**2**	**< 0.001**
	Diet × Sex	1.677	1	0.195
	Age × Sex	2.810	2	0.245
	Diet × Age × Sex	5.175	2	0.075
Nutritional	Wet weight	**5.990**	**1**	**0.014**
	Protein	**16.466**	**1**	**< 0.001**
	Lipid	0.000	1	0.996
	Carbohydrate	0.011	1	0.915
	Diet	3.046	1	0.081
	Age	3.217	2	0.200
	Sex	0.341	1	0.560
Methyl eugenol				
Intrinsic	Diet	**26.100**	**1**	**< 0.001**
	Age	**24.589**	**2**	**< 0.001**
	Diet × Age	**10.292**	**2**	**0.006**
Nutritional	Wet weight	1.134	1	0.287
	Protein	**3.872**	**1**	**0.049**
	Lipid	**4.42**	**1**	**0.036**
	Carbohydrate	1.476	1	0.224
	Diet	0.943	1	0.332
	Age	0.371	2	0.831

*Note* Bold values indicate significant terms

Flies fed a protein-rich diet that were 10- or 20-days-old were used to assess the effects of abiotic variables on the response of *B. dorsalis* to methyl eugenol. The minimum adequate model included the linear effects of temperature, humidity, and light intensity. Temperature, humidity, and light intensity had significant effects on fly response (Table 7). The response of *B. dorsalis* to methyl eugenol increases with temperature (Table 7). No flies are predicted to respond to methyl eugenol baited traps at temperatures below 23.0 °C, 95% CI [21.23, 24.67] (Fig. 7(ii)). Additionally, an increase in response of 0.3 flies is predicted for every 1 °C increase in temperature. Similarly, response increases with relative humidity (Table 6). No flies are predicted to respond at relative humidity levels below 45.7% RH, 95% CI [44.02, 47.46], with an increase of 0.8 flies predicted for every 10% RH increase in relative humidity. Light intensity also affected fly response, with response decreasing with higher light intensity (Table 6). No flies are predicted to respond at light intensities above 13 158.5, 95% CI [13156.78, 13160.22], and a decrease in response of 1.2 flies was predicted for every 1000 lx increase in light intensity.

**Table 7:** The effects of extrinsic factors on the response of *B. dorsalis* to two attractants

Attractant		Coefficient ± SE	χ2	df	*p*
Biolure					
Linear effects	Temperature	**3.372 ± 0.720**	**21.961**	**1**	**< 0.001**
	Humidity	**0.407 ± 0.111**	**13.508**	**1**	**< 0.001**
	Light intensity	**-0.001 ± 0.000**	**10.047**	**1**	**0.002**
	Temperature × Humidity	**-0.178 ± 0.004**	**13.177**	**1**	**< 0.001**
	Temperature × Light intensity	**0.000 ± 0.000**	**9.179**	**1**	**0.002**
	Humidity × Light intensity	**0.000 ± 0.000**	**10.474**	**1**	**0.001**
	Temperature × Humidity × Light intensity	**-0.000 ± 0.000**	**9.130**	**1**	**0.003**
Non-linear effects	Temperature × Temperature	**-0.053 ± 0.014**	**21.241**	**1**	**< 0.001**
	Humidity × Humidity	**-0.001 ± 0.000**	**13.515**	**1**	**< 0.001**
	Light intensity × Light intensity	0.000 ± 0.000	0.134	1	0.714
Methyl eugenol					
Linear effects	Temperature	**0.123 ± 0.020**	**37.208**	**1**	**< 0.001**
	Humidity	**0.020 ± 0.006**	**10.264**	**1**	**0.001**
	Light intensity	**− 0.000 ± 0.000**	**10.486**	**1**	**0.001**

*Note* Bold values indicate significant terms

### Lure Formulation Weight Change

**Fig. 8:**
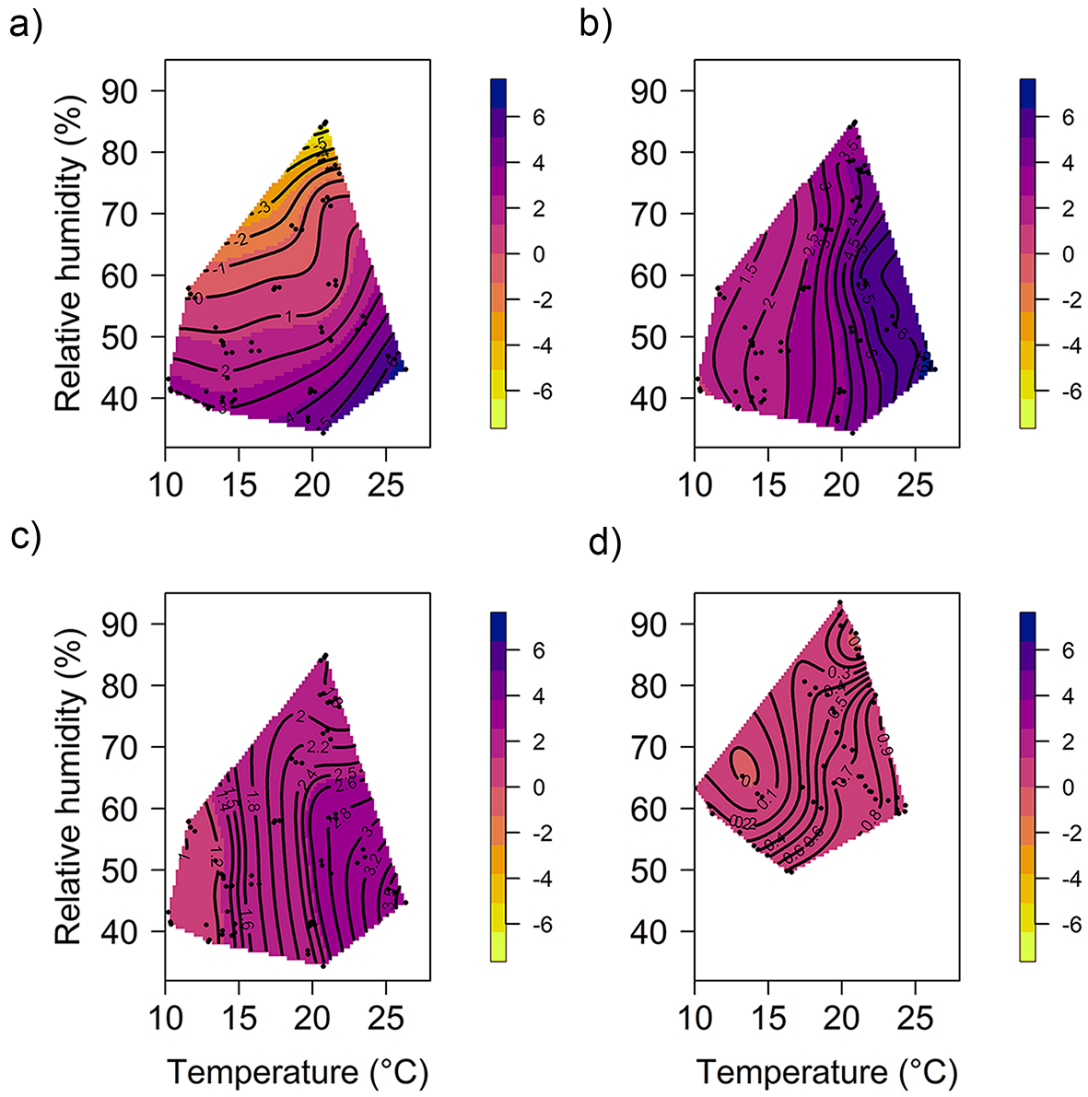
Response surface plot relating relative humidity and temperature to the weight change of (**a**) BioLure. (**b**) E.G.O PheroLure. (**c**) methyl eugenol. (**d**) CapiLure when placed in a yellow bucket trap and suspended in a citrus orchard for 30 days. The colour gradient indicates the percentage weight change of the lures

When tested in the field, temperature significantly affected weight loss from BioLure, E.G.O Pherolure, methyl eugenol and Trimedlure (Capilure) (Fig. 8(A, B, C, and D). In each case, weekly weight loss increased with temperature, with the effect being strongest in E.G.O Pherolure, with approximately 1% more lure formulation weight lost per week for each 1 °C increase in temperature (log-transformed data, linear mixed effects: coefficient = 0.016; c^2^ = 17.737, df = 1, *P* < 0.001). This was followed by Trimedlure (coefficient = 0.004, c^2^ = 15.04, *P* < 0.001), BioLure (coefficient = 0.295; c^2^ = 11.718, df = 1, *P* < 0.001) and methyl eugenol (coefficient = 0.007; c^2^ = 8.245, df = 1, *P* = 0.004). Trimedlure lost a small amount of weight as relative humidity decreased (coefficient = − 0.001, c^2^ = 8.74, *P* = 0.003). In addition, relative humidity interacted significantly with temperature to affect BioLure weight loss (coefficient = -0.006; c^2^ = 9.247, df = 1, *P* = 0.002); at high weekly average relative humidity and low weekly average temperature BioLure gained weight. Weekly average relative humidity did not affect weight loss from E.G.O Pherolure or methyl eugenol.

## Discussion

In this study, we used standardised methods with known numbers of flies in field cages that enabled us to compare how different species respond to commercially available lures under different biological and environmental conditions. The strength of this approach over field-based studies (Bali et al. 2021; Cornelius et al. 2000; Makumbe et al. 2017) is that our results give clear estimates of the relative number of flies within a tree canopy that will respond to a lure without being confounded by unknown population sizes or motivational states of individuals in a population. For example, recapture rates in tephritid mark-release-recapture studies vary widely, with rates ranging from 0.03 to 65%, depending on species, trap distance, and the attractant used (Reynolds et al. 2012; Manoukis et al. 2015). By using this approach, we have found that the response of each species to lures designed to target it are complex, with intrinsic, biological factors like sex, age, and diet being important determinants. However, even among individuals that are most responsive, physical environmental conditions need to be within a range that enable flies to respond to a trap containing the lure.

Contrary to expectations, there was no consistent bias towards the capture of females in traps baited with BioLure. In *C. cosyra* and *B. dorsalis*, sex had no significant effect on response by flies to traps baited with BioLure. Only in *C. capitata* was there an effect of sex when females were deprived of protein. Several studies have documented the female-bias of BioLure-baited trap captures for *C. capitata, C. cosyra* and *B. dorsalis* (Colacci et al. 2022; Leza et al. 2008; Manrakhan et al. 2017). However, the absence of a female-biased response to BioLure has also been observed. Peñarrubia-María et al. (2014) found that similar proportions of female and male *C. capitata* responded to BioLure in commercial citrus orchards. In another study there was a male-biased response of *C. capitata* to BioLure, which was linked to lower temperatures (Bali et al. 2021). Our results from *C. cosyra* and *B. dorsalis* suggest that attraction to protein (and thus food-based lures, such as BioLure) does not motivate females of these species as strongly as in *C. capitata*. *Ceratitis cosyra* females do not consume large quantities of protein in their diet (Malod et al. 2023), and this species has a lower reproductive output compared to *C. capitata* (Pogue et al. 2022). Similarly, *B. dorsalis* does not require large quantities of dietary protein to optimise its reproduction (Hou et al. 2020). The results of this study were obtained in semi-field cages with host trees that had no fruit. Females are known to group around host trees with ripe and semi-ripe fruit (Papadopoulos et al. 2001, 2003), with female-biased traps recommended to be placed in trees with ripe or ripening fruit (Lance and Gates 1994). As fruit growth in the host trees in this study was absent it is possible that the female selectivity of BioLure was reduced. Tephritid flies are attracted to the colour yellow (Prokopy and Owens 1983), and the use of yellow-coloured traps may have inadvertently attracted flies, regardless of the attractant within. Added to the restricted size of the test environment (field cages), this may explain why in some cases the control trap caught more flies than the experimental trap. However, the use of a control trap accounts for this type of inadvertent trap capture.

As expected, regardless of species, flies deprived of protein in their adult diet were more responsive to BioLure baited traps. However, in comparison with flies with high body protein, *C. capitata* and *B. dorsalis* flies with lower body protein content had a lower probability of response to BioLure. In other studies, protein-deprived flies were markedly more attracted to protein sources and food-based lures (Cohen and Voet 2002; Cornelius et al. 2000; Manrakhan and Lux 2008). Access to protein during the post-teneral stage plays an important role in sexual maturation and success in tephritids (Cangussu and Zucoloto 1995; Jácome et al. 1999; Shelly et al. 2002, 2005; Yuval et al. 1998), so there is a need for flies to seek out and consume protein. The discrimination threshold for protein detection is lowered in *C. capitata* that have been deprived of protein (Cangussu and Zucoloto 1995), which likely facilitates an increase in protein consumption to compensate for a nutritional imbalance. A lower response to BioLure in *C. capitata* and *B. dorsalis* that had a lower body protein content was thus unexpected. In *C. capitata*, protein consumption changes with age (Kouloussis et al. 2017). In this study, both age and protein content influenced the response of *C. capitata* and *B. dorsalis* to BioLure. It is thus possible that protein content fluctuates with age in these two species, possibly as a result of a change in protein consumption with age. Further research on the correlation between age, protein content and consumption in these species would be beneficial. Starvation decreases the expression of chemosensory receptors in *B. dorsalis* (Jin et al. 2017). Although protein deprivation increases the motivation of flies to seek out protein sources, this may have adverse effects on chemosensory processes that aid in perception and location of food sources. The effects of malnutrition on tephritid olfactory processes are often conflicting (Farhadian et al. 2012; Farhan et al. 2013; Root et al. 2011). More research is needed to clarify the effects of nutrition on the perception and response of fruit flies to food-based lures. High protein diets have shown to decrease overall activity and flight in female *Drosophila melanogaster* (Meigen), *(A) ludens* and *(B) tryoni* (Catterson et al. 2010; Fanson et al. 2013; Zou et al. 2011). However, male *D. melanogaster* activity decreases under low protein conditions (Catterson et al. 2010). It is possible that *(C) capitata* and *B. dorsalis* with low protein reserves had decreased response to BioLure as a result of a general decline in activity and flight. However, it is also possible that low protein reserves may have been a result of decreased muscle mass, which would impair flight activity. The effects of diet quality on activity and flight patterns appear to be species-specific. As such, additional research on the effects of nutritional composition on activity and flight performance in *C. capitata* and *B. dorsalis* would be beneficial.

In accordance with our hypothesis, young *C. cosyra* and *C. capitata*, that are still approaching maturity (two-, or 10-day-old males), were more responsive to male lures than mature flies. However, in this study the response of *B. dorsalis* to methyl eugenol was lowest in immature males. E.G.O PheroLure and Trimedlure attract mature and immature *C. capitata* males (Shelly et al. 2002; Shelly 2001). Immature males exposed to E.G.O PheroLure receive delayed mating benefits as they reach maturity (Shelly et al. 2002; Shelly 2001). The attraction of immature males is likely a result of delayed mating advantages they receive. However, unlike E.G.O PheroLure, the benefits of Trimedlure are short-lived and last no longer than 24 h for *C. capitata* (Shelly et al. 1996). Laboratory rearing increases the rate of sexual maturation in *C. capitata* (Vargas and Carey 1989*)*, with optimal mating occurring as young as three-days old (Liedo et al. 2002). It is likely that two-day-old male *C. capitata* and *C. cosyra* would also receive mating benefits from Trimedlure at this age. Feeding and attraction by males of some *Bactrocera* species, such as *B. zonata* and *B. dorsalis*, to methyl eugenol increases with age with a peak in response occurring at sexual maturity (around 10 days of age for laboratory-reared flies) (Rasool et al. 2023; Shelly 2020; Wee and Tan 2000; Wong et al. 1989). Methyl eugenol is bioconverted into the male sex pheromone, which is released upon sexual maturity (Shelly et al. 2008), enhancing the mating success of *B. dorsalis* (Shelly 2010; Haq et al. 2018). Although response to methyl eugenol increased with age, some immature flies still responded to methyl eugenol. It is not clear why immature *B. dorsalis* feed on methyl eugenol as they receive no mating benefits (Shelly et al. 2008). The methyl eugenol feeding may thus have benefits beyond the scope of sexual selection, with Hong and Nishida (1998) proposing that methyl eugenol feeding at young ages makes *B*. *dorsalis* distasteful to predators.

Although we predicted that lure response would increase with temperature, we found that these effects are complex and lure specific. Warmer temperatures resulted in increased responses to male lures and the response of *B. dorsalis* increased with temperature regardless of lure. However, we found that temperature plays little role in the response of *C. capitata* and *C. cosyra* to BioLure. Temperature is one of the main drivers of fruit fly demography (Duyck et al. 2004; Nyamukondiwa and Terblanche 2009; Pieterse et al. 2017), with fly activity and dispersal generally increasing in warmer conditions (Esterhuizen et al. 2014; Makumbe et al. 2020; Ye and Liu 2005). Furthermore, warmer temperatures support better sexual performance of male *C. capitata* (Weldon et al. 2022). Greater attraction to male lures at higher temperatures may thus be a result of increased activity, as well as a means for male fruit flies to further improve their reproductive potential. Unlike our findings, field studies show that captures of *C. capitata* in BioLure baited traps increase with temperature, and in accordance with our findings, trap captures for *B. dorsalis* increased with temperature (Manrakhan et al. 2017). However, this was more strongly associated with the ripening of nearby citrus. *Ceratitis capitata* and *C. cosyra* have wide ranges in thermal tolerance and desiccation resistance (Nyamukondiwa and Terblanche 2009; Pullock et al. 2023; Weldon et al. 2016, 2018). Both species are thus likely to forage for food resources under a wide range of environmental conditions. Thus, in the absence of ovipositional drivers, such as ripening host fruits, it appears that temperature alone has little effect on the response of *C. capitata* and *C. cosyra* to BioLure for the ranges observed in this study.

Our data on the response of *B. dorsalis* to lures concur with studies that measured flight activity under varying temperatures (Makumbe et al. 2020; Motswagole et al. 2019). We found that peak response of *B. dorsalis* occurred at 29.1 and 23.0 °C to BioLure and methyl eugenol, respectively, which approximates the optimal flight temperature of *B. dorsalis* (Makumbe et al. 2020). Based on model predictions, BioLure response was restricted at temperatures lower than 17.9 °C or above 40.3 °C. Although temperatures as high as 40.3 °C was not observed in this study it was found that trap capture started to decline above 29.1 °C. The upper critical thermal limit for *B. dorsalis* is approximately 46 °C, with flight activity restricted above 38 °C (Makumbe et al. 2020; Motswagole et al. 2019). Similarly, the lower critical thermal limit for *B. dorsalis* is 9 °C, with flight restricted at temperatures below 12 °C (Makumbe et al. 2020; Motswagole et al. 2019). Higher responses of *B. dorsalis* to lures at warmer temperatures are likely to be a result of their increased flight performance and although *B. dorsalis* can fly between 12 and 17 °C, it is likely that flies will prioritise warming behaviours such as grooming over responding to lures at these temperatures. However, higher lure response by some species as temperatures increase cannot always be separated from the potential rate of lure release from dispensers. The higher rate of weight loss from E.G.O Pherolure and methyl eugenol dispensers that we recorded as field temperatures increase also coincides with higher response (especially by males) of their target species. While the lure dispensers differed between tests for response and weight change, the limited change in Trimedlure dispenser weight with temperature is one example where response may mostly be driven by fly activity.

Relative humidity had little effect on lure response for *C. capitata* and *C. cosyra* for the ranges observed. This concurs with findings by Manrakhan et al. (2017) when investigating lure response by *C. capitata, C. cosyra* and *B. dorsalis*. Relative humidity affects the release rate of volatile lures and influences fruit fly trap captures (Harris et al. 1971). Our results further show that the effect of relative humidity on lure weight loss and volatilisation is lure specific. Relative humidity only affected the lure response of *B. dorsalis*, although not consistently. Decreased response to BioLure at higher relative humidity corresponded with weight gain of the lure. This indicates that there was less lure present in the air, which would make the lure harder to detect by *B. dorsalis*. Low relative humidity is often correlated with high temperatures. The predicted peak response of *B. dorsalis* to BioLure at low relative humidities thus further indicates that high temperatures elicit a greater response to BioLure in this species. However, response of *B. dorsalis* to methyl eugenol increased with humidity, with humidity having no effect on lure weight. This indicates that the increase in response is not due to the availability of the lure in the air and is rather linked to the motivational state of the fly. *Bactrocera dorsalis* prefers hot and humid climates, which may explain why it remains active and is inclined to respond to methyl eugenol under higher humidity conditions (De Meyer et al. 2010). The interspecific variation with regards to the effect of humidity may also be a result of interspecific variation in preferred climate, with *C. capitata* being adapted to drier environments (Duyck et al. 2006).

Our results show that trap capture is variable and depends on both fly physiology and the abiotic environment. Natural sources of protein, such as animal excreta, pollen and decaying organic matter are consumed by fruit flies to obtain their nutritional needs (Drew and Yuval 1999). As flies with protein-rich diets had lower responses to Biolure, the abundance of natural sources of protein will decrease the efficacy of BioLure baited traps, causing monitoring programmes to underestimate fruit fly populations. This study shows that approximately 6% of the female *C. capitata* population within a tree canopy will respond to BioLure if they have had adequate access to protein, whereas 19% of females respond when their access to protein has been restricted. Similarly, for *C. cosyra* capture probability ranges from approximately 1 to 4% of females for protein-rich and protein-deprived flies respectively. In *B. dorsalis*, female trap captures for BioLure baited traps range from 4 to 23%, depending on adult diet. As such, monitoring programmes should consider the availability of natural sources of protein, and orchard sanitation, when estimating fruit fly populations. Natural sources of protein primarily stem from rotting fruit and leaf surface bacteria (Hendrichs and Hendrichs 1990; Drew et al. 1983). Orchard sanitation may limit some natural sources of protein. However, leaf surface bacteria abundance in the field is largely out of the control of producers. Different leaf surface bacterial taxa are enrichened under different climatic conditions (Li et al. 2022). This prevents the association of bacterial protein sources with particular environmental conditions. The lure response range observed in this study thus provides minimum and maximum population estimates under protein sufficient and protein insufficient environments. Additionally, monitoring programmes using BioLure need to acknowledge that even under ideal physiological conditions, a lower proportion of *C. cosyra* respond to BioLure than *C. capitata* and *B. dorsalis*.

We found that the response of *B. dorsalis* to BioLure is highest at roughly 29 °C, with approximately 40% of the population responding and that even under ideal conditions, no flies are likely to respond below 17.9 °C, or above 40.3 °C despite fly presence in the area. Beyond these temperatures, population estimates from trap captures will be highly inaccurate. Under ideal conditions, up to 72% of male *C. capitata* responded to E.G.O PheroLure, with an average additional 0.6 flies responding for every 1 °C increase in temperature. Under optimal conditions, up to 40% of male *C. capitata* responded to Trimedlure. However, males are unlikely to respond at temperatures below 12.2 °C or above 37.8 °C. This indicates that monitoring programmes using Trimedlure may be less sensitive to *C. capitata* population changes than those that are baited with E.G.O PheroLure. *Bactrocera dorsalis* males are less likely to respond to methyl eugenol baited traps below 23.0 °C, with an average increase in response of 1.2 flies per 1 °C increase. These results highlight the need to account for temperature variation in trapping surveys and monitoring programmes. Although, the minimum lure response threshold differs per species and lure, these results show that temperatures below 12 °C seldom result in any trap captures regardless of species or presence of nearby flies. Taking temperature variation into account is thus particularly important for traps that are set up in winter, or in higher elevations as they are more likely to underestimate fruit fly populations. Models based on the thermal reaction norm of the probability of fly lure response from our study could be combined with records of daily temperature over a period of trapping (usually one or two weeks) to relate trap captures on a flies per trap per day (FTD) basis to actual fly abundance. This mechanistic approach is applied to evaluate climate suitability for species (e.g., Kearney and Porter 2009; Buckley et al. 2010), so it can be extended to relate trap numbers to the duration of a trapping period suitable for fruit fly response (i.e., its activity niche (Arnan and Blüthgen 2015). While this does not account for the motivational state of the flies, it would provide a better basis for interpreting trap capture records than is currently the case. Counts of FTD integrate time, space, temperature and the range of biotic variables explored in our study so it is only an indirect and inaccurate measure of fly abundance, and thus is an unreliable indicator of presence when populations are small (Papadopoulos et al. 2013) or flies are unlikely to respond.

Reliable monitoring systems are crucial for the management of fruit fly pests. In this study, we found that fly physiology and the environment can significantly impact the efficacy of commercially used fruit fly lures. More specifically, flies with access to protein are less responsive to BioLure baited traps. We found that BioLure is not exclusively female-biased and that the effect of age on the response of fruit flies to male lures is species specific. Importantly, we found that lower temperatures often, but not always, result in a decreased lure response in tephritid fruit flies. We also found that warmer temperatures result in greater weight loss of lures. Trapping surveys provide important information to growers about fruit fly pest populations, which are used to establish and guide threshold management systems. Taken together, this research shows that trap captures – and the action thresholds they dictate – need to take physiological and environmental variation into account to increase trap sensitivity and accuracy for fruit fly management.

## Electronic Supplementary Material

Below is the link to the electronic supplementary material.

Supplementary Material 1

## Data Availability

The experimental data that support the findings of this study are publicly available. This data can be found here: https://doi.org/10.25403/UPresearchdata.25065908.
