# Supplementary material for: Effects of Physiological Status and Environmental Factors on the Lure Responses of Three Pest Fruit Fly Species (Diptera: Tephritidae)
Source: J Chem Ecol. 2024 Jul 8;50(11):679–700. doi: 10.1007/s10886-024-01516-8 (PMC11543777; doi:10.1007/s10886-024-01516-8)
Supplement: Supplementary file 1 — Supplementary Material 1 [file 10886_2024_1516_MOESM1_ESM.docx]

**Electronic supplementary material for:**

**EFFECTS OF NUTRITIONAL RESERVES, DIET AND WEATHER ON LURE RESPONSES OF THREE PEST FRUIT FLY SPECIES (DIPTERA: TEPHRITIDAE)**

TANIA POGUE^1^, KEVIN MALOD^1,2^ AND CHRISTOPHER W. WELDON^1*^

1 Department of Zoology and Entomology, University of Pretoria, Private Bag X20, Hatfield

0028, South Africa.

2 Current address: Department of Conservation Ecology and Entomology, Faculty of

AgriSciences, Stellenbosch University, Stellenbosch, South Africa

*Corresponding author: cwweldon@zoology.up.ac.za

ORCiD IDs:

Pogue: 0000-0002-7645-7109

Malod: 0000-0002-6811-2717

Weldon: 0000-0002-9897-2689


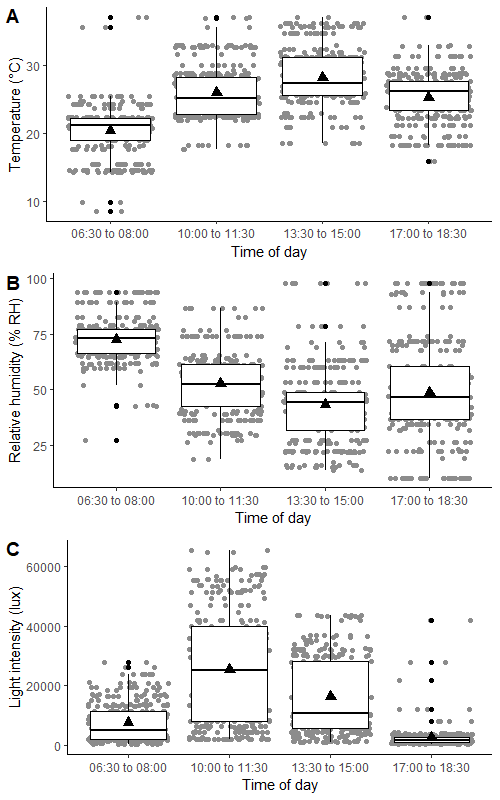


**Figure S1.** (A) Temperatures (°C), (B) relative humidities (% RH), and (C) light intensities (lux) recorded at four different times of the day (06:30 to 08:00, 10:00 to 11:30, 13:30 to 15:00, and 17:00 to 18:30) within a semi-field shade house during tephritid fruit fly lure response testing. Black triangles represent the mean value.

**
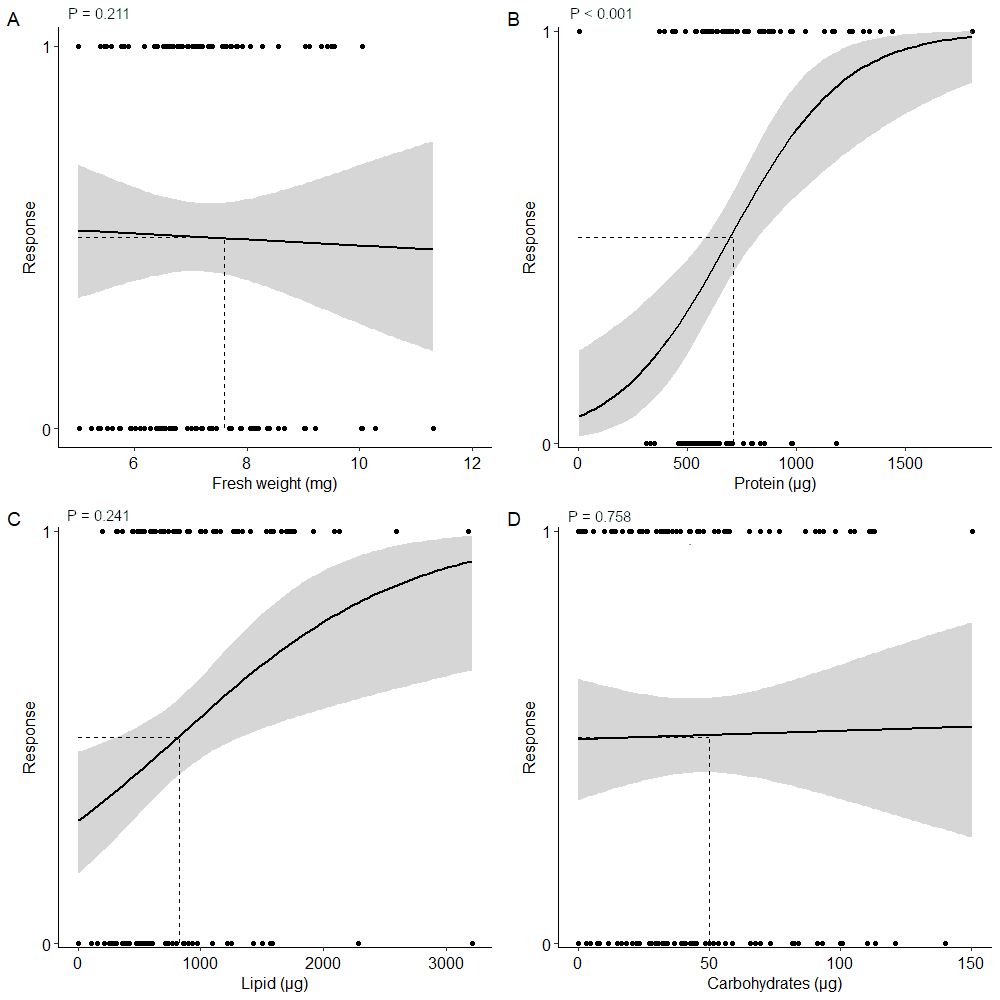
**

**Figure S2.** Probability that *C. capitata* responds to BioLure at varying (A) body weights (mg), (B) total body protein content (µg), (C) total body lipid content (µg), and (D) total body carbohydrate content (µg). Flies that responded to BioLure are coded as 1 and flies that were unresponsive are coded as 0. Trendlines, 95% confidence interval bands, and the equation of the relationship between factors are shown. The dashed line represents the value at which there is a 50% probability of response to BioLure (R_50_).


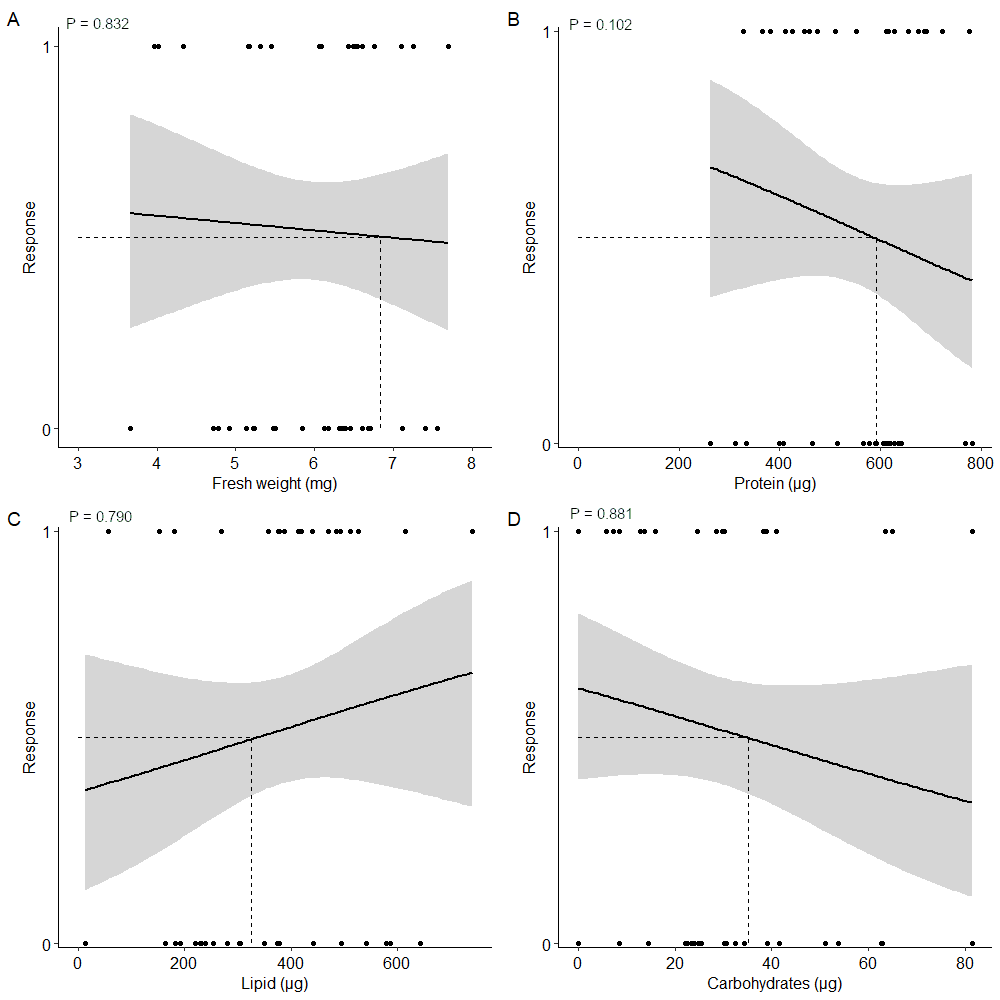


**Figure S3.** Probability that *C. capitata* responds to E.G.O PheroLure at varying (A) body weights (mg), (B) total body protein content (µg), (C) total body lipid content (µg), and (D) total body carbohydrate content (µg). Flies that responded to E.G.O PheroLure are coded as 1 and flies that were unresponsive are coded as 0. Trendlines, 95% confidence interval bands, and the equation of the relationship between factors are shown. The dashed line represents the value at which there is a 50% probability of response to BioLure (R_50_).


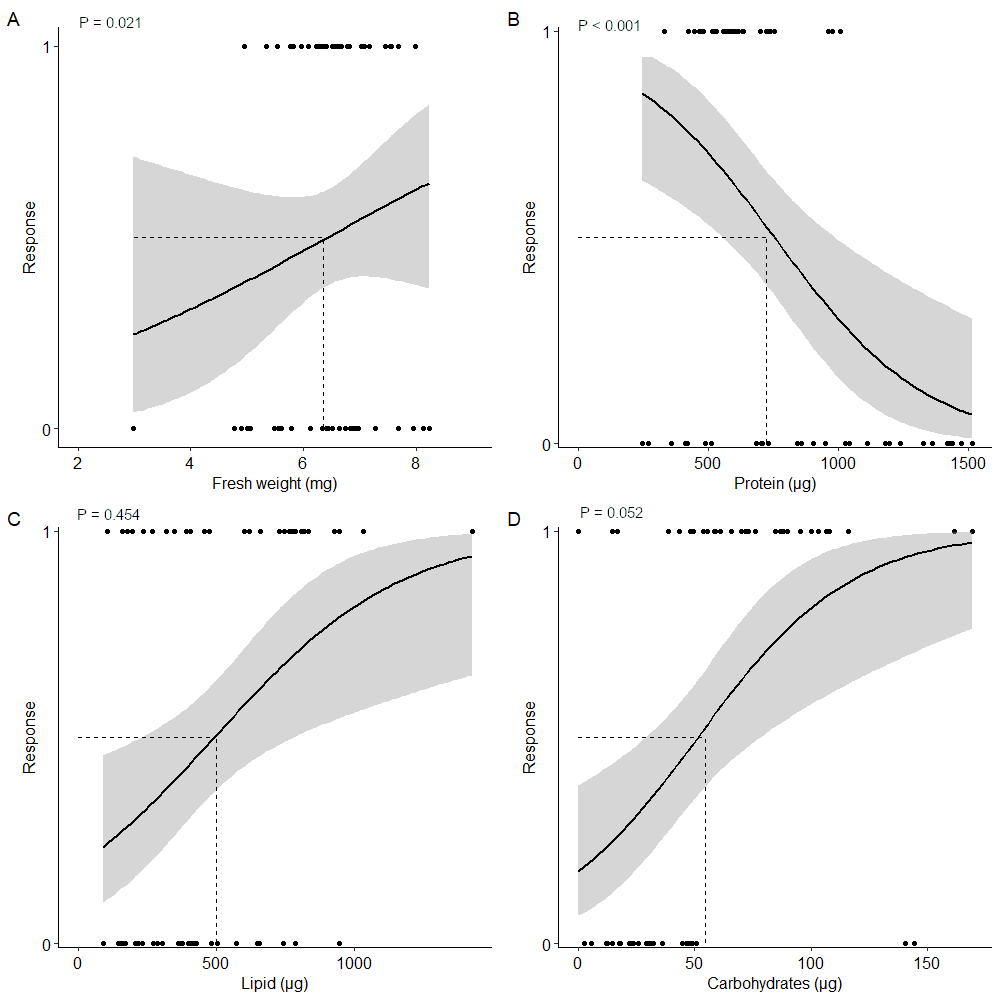


**Figure S4.** Probability that *C. capitata* responds to trimedlure at varying (A) body weights (mg), (B) total body protein content (µg), (C) total body lipid content (µg), and (D) total body carbohydrate content (µg). Flies that responded to trimedlure are coded as 1 and flies that were unresponsive are coded as 0. Trendlines, 95% confidence interval bands, and the equation of the relationship between factors are shown. The dashed line represents the value at which there is a 50% probability of response to trimedlure (R_50_).

**
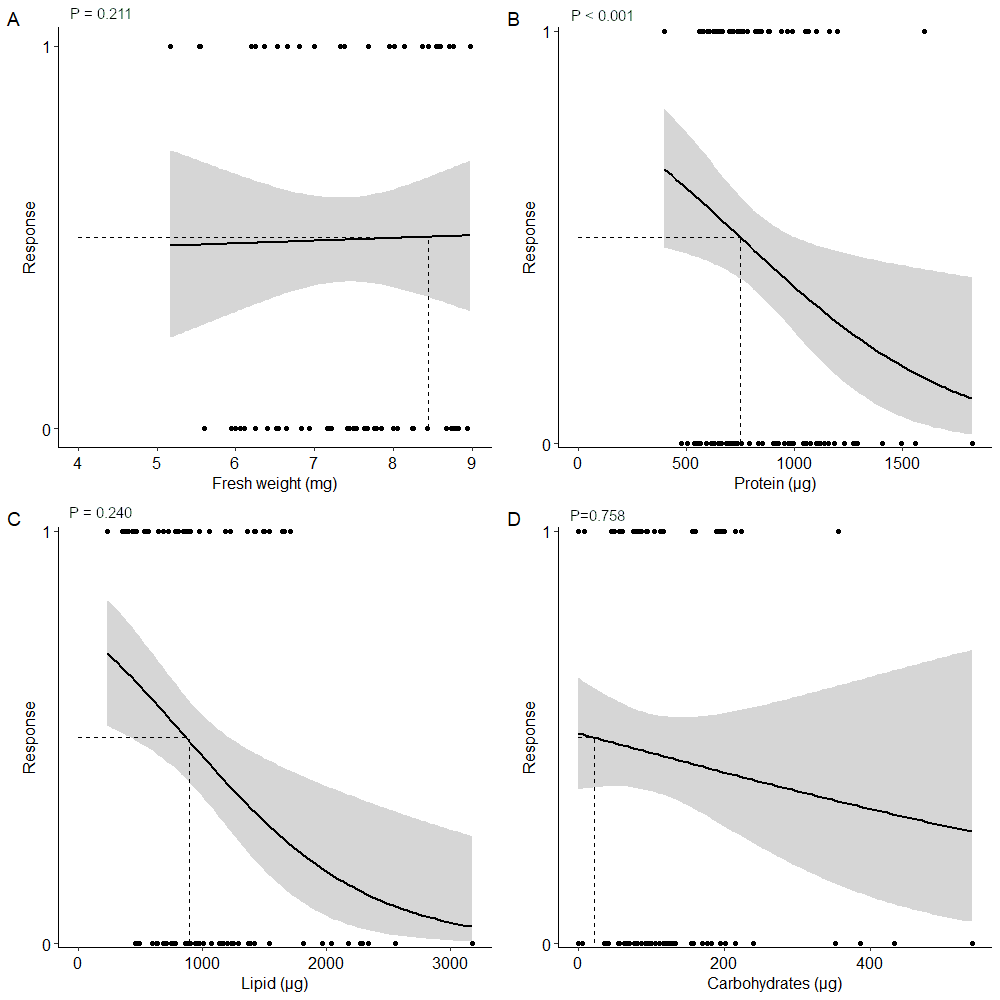
**

**Figure S5.** Probability that *C. cosyra* responds to BioLure at varying (A) body weights (mg), (B) total body protein content (µg), (C) total body lipid content (µg), and (D) total body carbohydrate content (µg). Flies that responded to BioLure are coded as 1 and flies that were unresponsive are coded as 0. Trendlines, 95% confidence interval bands, and the equation of the relationship between factors are shown. The dashed line represents the value at which there is a 50% probability of response to BioLure (R_50_).

**
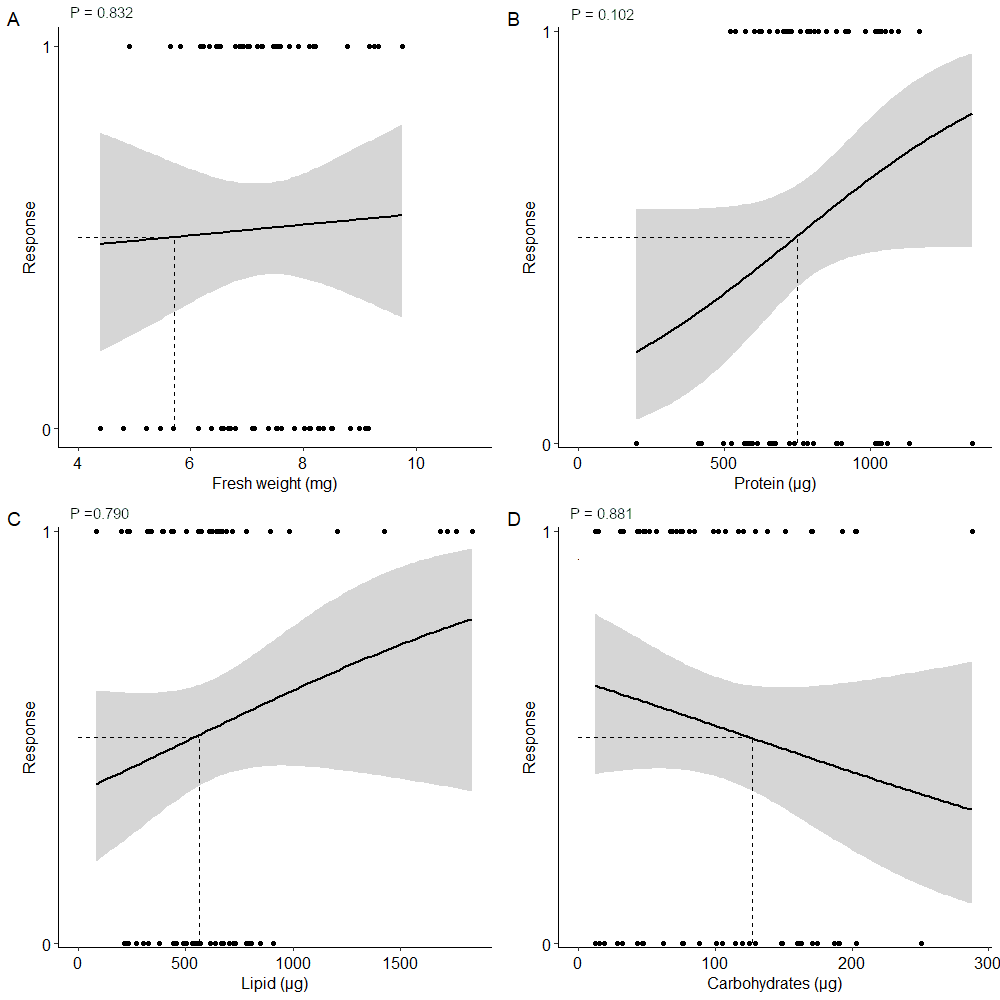
**

**Figure S6.** Probability that *C. cosyra* responds to E.G.O PheroLure at varying (A) body weights (mg), (B) total body protein content (µg), (C) total body lipid content (µg), and (D) total body carbohydrate content (µg). Flies that responded to E.G.O PheroLure are coded as 1 and flies that were unresponsive are coded as 0. Trendlines, 95% confidence interval bands, and the equation of the relationship between factors are shown. The dashed line represents the value at which there is a 50% probability of response to BioLure (R_50_).

**
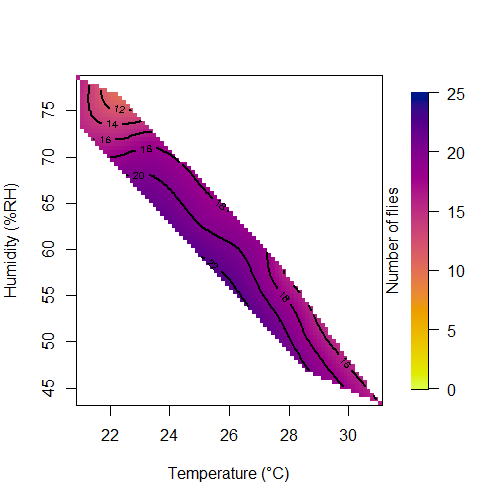
**

**Figure S7.** Response surface plot relating relative humidity and temperature to the trap capture of the most responsive experimental groups of *C. cosyra* (two- or ten-day old males fed either protein rich, or protein deprived diets) when 25 flies of each group were given 90 minutes to respond to a E.G.O Pherolure baited yellow bucket trap in a semi-field cage. Accidental trap capture was accounted for by using an unbaited yellow bucket trap, with the number of flies caught in the control trap subtracted from those caught in the baited trap. The colour gradient indicates how many flies were caught.

**
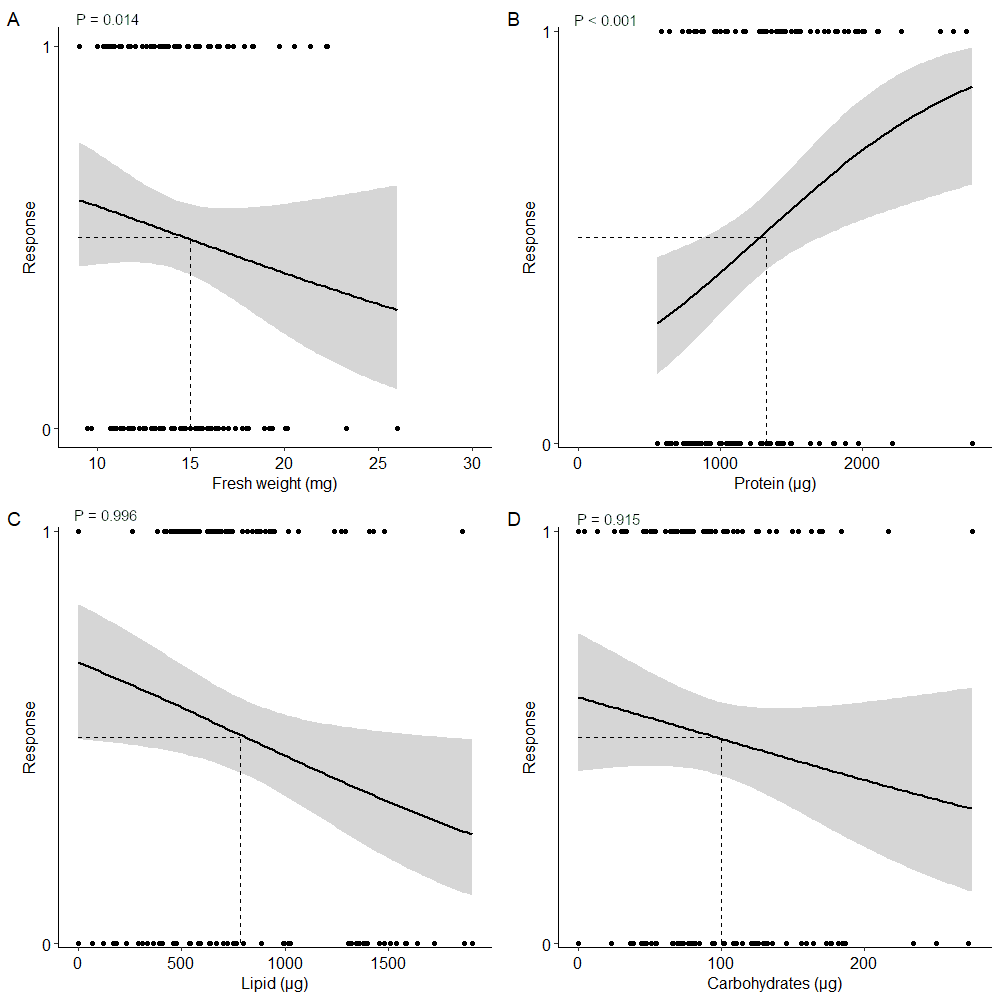
**

**Figure S8.** Probability that *B. dorsalis* responds to BioLure at varying (A) body weights (mg), (B) total body protein content (µg), (C) total body lipid content (µg), and (D) total body carbohydrate content (µg). Flies that responded to BioLure are coded as 1 and flies that were unresponsive are coded as 0. Trendlines, 95% confidence interval bands, and the equation of the relationship between factors are shown. The dashed line represents the value at which there is a 50% probability of response to BioLure (R_50_).

**
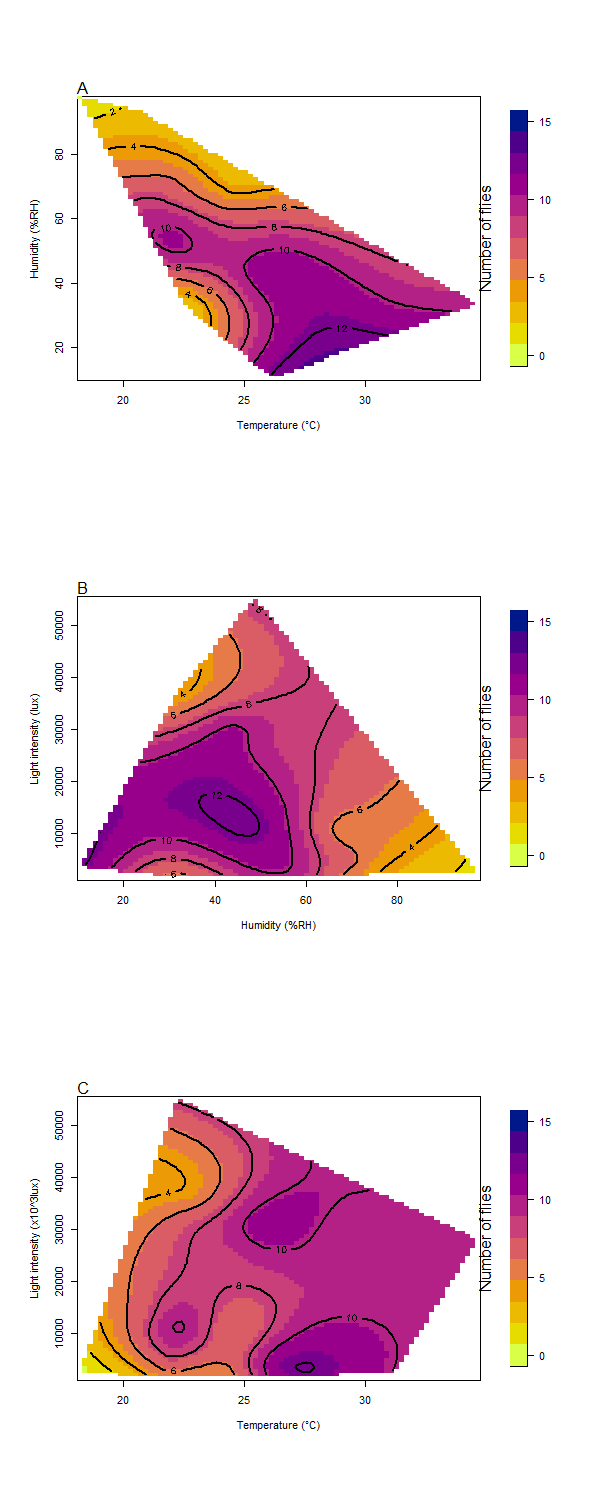
**

**Figure S9.** Trap captures as a function of relative humidity and temperature in the most responsive experimental groups of *B. dorsalis* (ten-day old males and females fed a protein deprived diet) when 25 flies of each group were given 90 minutes to respond to a Biolure baited yellow bucket trap in a semi-field cage. Accidental trap capture was accounted for by using an unbaited yellow bucket trap, with the number of flies caught in the control trap subtracted from those caught in the baited trap. The colour gradient indicates how many flies were caught.

**
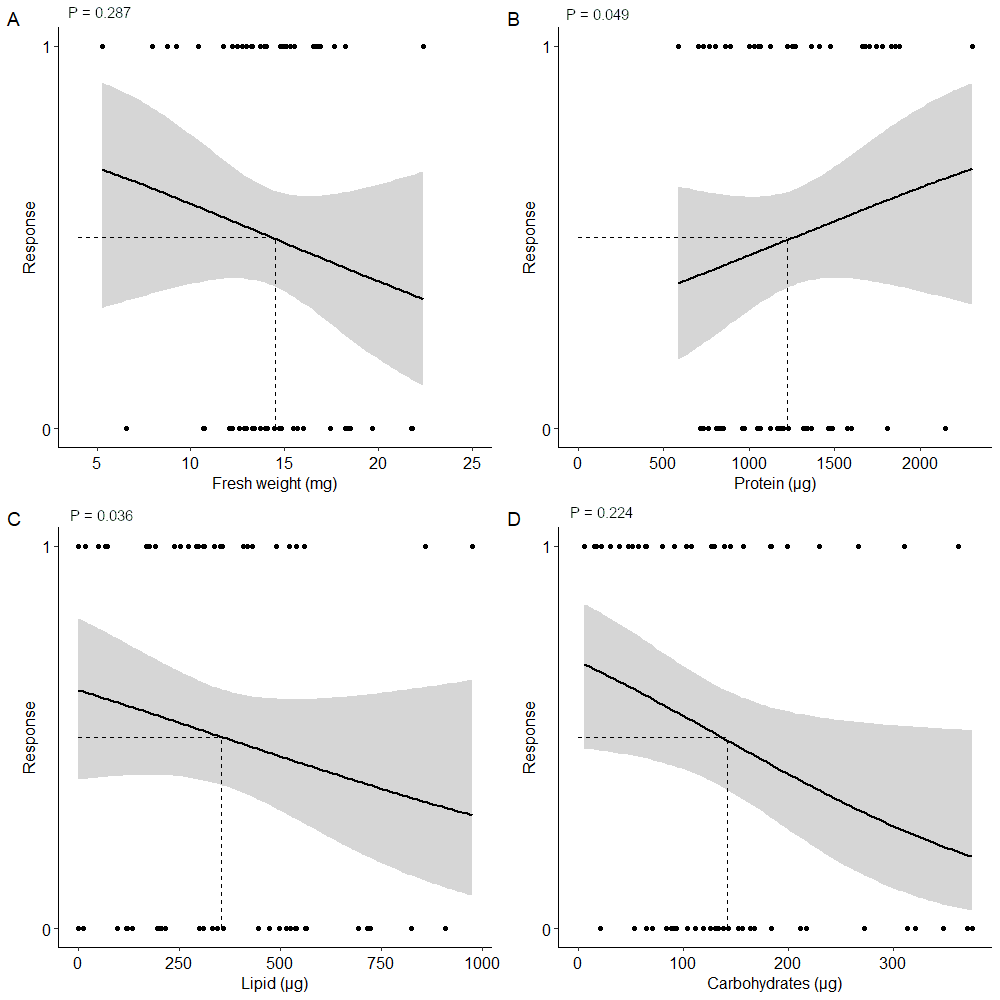
**

**Figure S10.** Probability that *B. dorsalis* responds to methyl eugenol at varying (A) body weights (mg), (B) total body protein content (µg), (C) total body lipid content (µg), and (D) total body carbohydrate content (µg). Flies that responded to methyl eugenol are coded as 1 and flies that were unresponsive are coded as 0. Trendlines, 95% confidence interval bands, and the equation of the relationship between factors are shown. The dashed line represents the value at which there is a 50% probability of response to BioLure (R_50_).
